# PM_2.5_, Fine Particulate Matter: A Novel Player in the Epithelial-Mesenchymal Transition?

**DOI:** 10.3389/fphys.2019.01404

**Published:** 2019-11-29

**Authors:** Zihan Xu, Wenjun Ding, Xiaobei Deng

**Affiliations:** ^1^Faculty of Public Health, Shanghai Jiao Tong University School of Medicine, Shanghai, China; ^2^Laboratory of Environment and Health, College of Life Sciences, University of Chinese Academy of Sciences, Beijing, China

**Keywords:** PM_2.5_, epithelial-mesenchymal transition, cancer, fibrosis, signaling pathway, molecular toxicology, air pollution, environment and human health

## Abstract

Epithelial-mesenchymal transition (EMT) refers to the conversion of epithelial cells to mesenchymal phenotype, which endows the epithelial cells with enhanced migration, invasion, and extracellular matrix production abilities. These characteristics link EMT with the pathogenesis of organ fibrosis and cancer progression. Recent studies have preliminarily established that fine particulate matter with an aerodynamic diameter of less than 2.5 μm (PM_2.5_) is correlated with EMT initiation. In this pathological process, PM_2.5_ particles, excessive reactive oxygen species (ROS) derived from PM_2.5_, and certain components in PM_2.5_, such as ions and polyaromatic hydrocarbons (PAHs), have been implicated as potential EMT mediators that are linked to the activation of transforming growth factor β (TGF-β)/SMADs, NF-κB, growth factor (GF)/extracellular signal-regulated protein kinase (ERK), GF/phosphatidylinositol 3-kinase (PI3K)/Akt, wingless/integrated (Wnt)/β-catenin, Notch, Hedgehog, high mobility group box B1 (HMGB1)-receptor for advanced glycation end-products (RAGE), and aryl hydrocarbon receptor (AHR) signaling cascades and to cytoskeleton rearrangement. These pathways directly and indirectly transduce pro-EMT signals that regulate EMT-related gene expression in epithelial cells, finally inducing the characteristic alterations in morphology and functions of epithelia. In addition, novel associations between autophagy, ATP citrate lyase (ACLY), and exosomes with PM_2.5_-induced EMT have also been summarized. However, some debates and paradoxes remain to be consolidated. This review discusses the potential molecular mechanisms underlying PM_2.5_-induced EMT, which might account for the latent role of PM_2.5_ in cancer progression and fibrogenesis.

## Introduction

Air pollution, especially that of developing countries, has become increasingly severe with the process of industrialization. Even before atmospheric brown cloud (ABC) was first observed in Asia, ambient particulate matter (PM) has been widely regarded as one of the major airborne pollutants. PM is composed of solids and/or liquids suspended in the atmosphere with a large variation in size. PM can be classified into four different categories according to aerodynamic diameters: total suspended particulates (TSPs); inhalable particles with an aerodynamic diameter less than 10 μm (PM_10_); fine PM with an aerodynamic diameter less than 2.5 μm (PM_2.5_); and ultrafine particles (PM_0.1_). Owing to the relatively high dispersity and small diameter, PM_2.5_ can be inhaled and deposited in airways and alveoli and can even penetrate into the bloodstream, systemically spreading and inducing damage to multiple systems and organs (Wang et al., 2013; Falcon-Rodriguez et al., 2016; Liu et al., 2019). PM_2.5_ is derived from industrial and vehicle emissions, mineral dust, biomass incineration, and other diverse sources; thus, the composition of PM_2.5_ is extremely complex (Belis et al., 2013; Falcon-Rodriguez et al., 2016). Transitional metals, soluble salts, organic/elemental carbon, and microbes that adhere to PM_2.5_ particles contribute to the toxic effects of PM_2.5_ including inflammation, DNA damage, mutations, reactive oxygen species (ROS) accumulation, and mitochondria dysfunction. All these abnormal intracellular events are highly correlated with the pathogenesis and progression of a series of human diseases (Ghio et al., 2012; Valavanidis et al., 2013; Feng et al., 2016; Qi et al., 2019). Outdoor PM_2.5_ exposure has been listed as the fifth most influential risk factor for global death, which caused 4.2 million deaths in 2015 (Schraufnagel et al., 2018). Besides, epidemiological studies have shown that PM_2.5_ exposure was highly correlated with the morbidity and mortality of respiratory and cardiovascular diseases. Maternal exposure to PM_2.5_ is also linked to an increased risk of airway disease development in offspring (Saha et al., 2018; Sanyal et al., 2018).

Epithelial-mesenchymal transition (EMT) is a process through which epithelial cells lose their apical-basal polarity and acquire the mesenchymal phenotype (Katsuno et al., 2013; Chaffer et al., 2016). A series of underlying molecular events, especially the repression of E-cadherin expression, the upregulation of N-cadherin and vimentin, and the disruption of adherin junctions, have been uncovered (Kourtidis et al., 2017; Miyazono et al., 2018). Other significant molecular features underlying EMT include cytoskeleton remodeling, such as actin stress fiber formation, and induction of mesenchymal biomarkers, including α-smooth muscle actin (α-SMA) and fibronectin (Kalluri and Weinberg, 2009). As EMT endows the epithelia with the potential of myofibroblast differentiation, increased motility, migration and invasion ability, this cellular event, if abnormally activated, will inevitably mediate the progression of organ fibrosis and cancer metastasis by facilitating the secretion of the extracellular matrix components or the dissociation of neoplastic cells from primary tumors and the subsequent intravasation (Stone et al., 2016; Yan et al., 2018; Lu and Kang, 2019). Furthermore, the enhanced anti-apoptosis and drug detoxification characteristics of cancer cells that have undergone EMT also result in drug resistance in cancer chemotherapy (Santamaria et al., 2019).

To date, three EMT subtypes have been determined based on their biological functions in tissue development and pathogenesis. Type 1 EMT is associated with the formation and implantation of embryo together with organ development. Type 2 EMT functions in wound healing by contributing to fibroblast generation and extracellular matrix production. Type 3 EMT refers to the phenotypic transformation in neoplastic epithelial cells, which enhances anti-apoptosis and invasion abilities (Kalluri, 2009; Kalluri and Weinberg, 2009; Zou et al., 2017).

Although massive statistical analyses have established the causal link between environmental PM_2.5_ exposure and morbidity/mortality of multiple cancers as well as fibrotic diseases, and “outdoor air pollution, particulate matter in” has been indexed as one of 120 primary carcinogens, the exact in-depth mechanisms at the cellular or microscopic level are still largely unknown (Kim et al., 2018; Sese et al., 2018; Cheng et al., 2019; International Agency for Research on Cancer, 2019). As these diseases are currently largely irreversible and lethal, targeted approaches to prevent their onset should be developed. Current research has demonstrated that acute or chronic PM_2.5_ exposure elicited the expression of characteristic EMT markers and EMT-related transcription factors (TFs) in the epithelia (Wei et al., 2017; Chi et al., 2018; Xu et al., 2019a). Similar altered expression patterns have also been detected in the lungs after mice were exposed to PM_2.5_ (Fu et al., 2019). Hence, it is possible that PM_2.5_ might serve as an exogenous promoter of fibrosis and cancer by initiating abnormal EMT in corresponding target epithelial tissues and cells. Although the molecular toxicology of PM_2.5_-induced EMT has not been well studied, on the basis of existing publications, this review aims to describe validated or potential molecular processes, especially the signaling pathways, participating in the action of PM_2.5_ on pro-fibrotic or neoplastic EMT.

## Overview of the Molecular Players in Epithelial-Mesenchymal Transition

### Key Transcription Factors

The loss of E-cadherin is a typical EMT hallmark. The TFs that control the expression of E-cadherin include the Zn-finger proteins Snail and Slug, the two-handed Zn-finger proteins ZEB1 and ZEB2, and the basic helix-loop-helix family protein TCF and Twist have also been identified (Guaita et al., 2002; Burger et al., 2017). These TFs directly bind to 5′ E-boxes of the human E-cadherin promoter and further silence the transcription of CDH1 (Batlle et al., 2000; Guaita et al., 2002; Vesuna et al., 2008). Lymphoid enhancer-binding factor-1 (LEF-1), a member of TCF/LEF family, also regulates EMT by recruiting the coactivator β-catenin to enhancers (Gilles et al., 2003; Santiago et al., 2017). Interactions and crosstalk between TFs have also been extensively recognized. Snail can induce the expression of ZEB1/2, Twist, Slug, and LEF-1 (Guaita et al., 2002; Dave et al., 2011; Cevenini et al., 2018). Twist can serve as the transcriptional initiator of Slug and ZEB1, whereas TCF-4 functions indirectly *via* ZEB1 (Taube et al., 2010; Sanchez-Tillo et al., 2011). LEF-1 can also exert its pro-EMT ability indirectly by upregulating Slug expression (Lambertini et al., 2010). In addition, a member of the high-mobility group (HMG) protein family, the transforming growth factor β (TGF-β)/SMAD2/3-dependent TF HMGA2, induces the expression of Snail, Slug, and Twist and mediates EMT (Thuault et al., 2006, 2008). NF-κB, a versatile TF involved in the regulation of proliferation, adhesion, and inflammation, executes its pro-EMT ability *via* Snail, Slug, and ZEB1/2 (Min et al., 2008; Sarkar et al., 2008; Techasen et al., 2012). Key TFs related to EMT and their transcriptional interactions are roughly summarized in Table 1.

**Table 1:** Positive transcriptional regulation among pro-EMT transcription factors.

Transcriptional inducers	Effectors	Reference
Snail	Slug	Cevenini et al. (2018)
	Twist	Cevenini et al. (2018)
	ZEB1	Dave et al. (2011)
	ZEB2	Cevenini et al. (2018)
	LEF-1	Guaita et al. (2002)
LEF-1	Slug	Lambertini et al. (2010)
Twist	Slug	Taube et al. (2010)
	ZEB1	Taube et al. (2010)
TCF-4	ZEB1	Sanchez-Tillo et al. (2011)
HMGA2	Snail	Thuault et al. (2008)
	Slug	Thuault et al. (2006)
	Twist	Thuault et al. (2006)
NF-κB	Snail	Sarkar et al. (2008)
	Slug	Storci et al. (2010)
	ZEB1	Min et al. (2008)
	ZEB2	Min et al. (2008)

*EMT, epithelial-mesenchymal transition*.

### Pro-Epithelial-Mesenchymal Transition Signaling Cascades

TGF-β is considered as the most powerful initiator of EMT. TGF-β can directly and positively regulate mesenchymal markers such as α-SMA and type I collagen (COL1) and is also a potent regulator of cytoskeleton remodeling (Zavadil and Bottinger, 2005; Fernandez and Eickelberg, 2012). The type II TGF-β receptor (TβRII) is initially recruited by activated TGF-β and forms a hetero-tetrameric complex with the type I TGF-β receptor (TβRI) by phosphorylating the GS domain of TβRI (Miyazawa et al., 2002; Huang and Chen, 2012; Vander Ark et al., 2018). SMAD2 and SMAD3, receptor-activated SMAD proteins (R-SMADs), are cytoplasmic mediators of TGF-β signaling whose recruitment and phosphorylation by TβRI result in their translocation to the nucleus in assistance of SMAD4 (Fabregat and Caballero-Diaz, 2018). SMAD2/3/4 together with other coactivators activates HMGA2, Snail, Slug, and ZEB1/2 expression, which further initiates the phenotypic transformation of EMT (Thuault et al., 2006; Katsuno et al., 2013). Another set of SMAD signaling cascades consisting of bone morphogenetic protein receptors (BMPRs) and the SMAD 1/5/8 also enhances Slug and Twist expression (McCormack et al., 2013; Li et al., 2014; Ahn et al., 2016; Wang et al., 2016). SMAD6 and SMAD7, two inhibitory SMADs (I-SMADs), participate in the inhibition of signal transduction *via* competitive binding with TβRI and BMPRI (Itoh and ten Dijke, 2007; Gonzalez-Nunez et al., 2013; Koganti et al., 2018). There are also non-canonical TGF-β cascades linked with EMT, in which TβRII phosphorylates Par6 and further modulates activity of RhoA *via* ubiquitination mediated by Smurf1, resulting in the loss of tight junction and EMT (Choi et al., 2017; Simeone et al., 2018).

β-Catenin is the main effector of Wnt pathway. The cytoplasmic accumulation of β-catenin after activation of Wnt signaling, or the release of β-catenin due to the loss of E-cadherin, which allow the interactions between β-catenin and LEF-1, can induce the upregulation of Snail, Slug, Twist and mesenchymal markers such as fibronectin (Guo et al., 2012; Ghahhari and Babashah, 2015). When the Wnt ligands bind to Frizzle and LDL receptor-related protein-5/6 (LRP5/6) receptors, extracellular signals are transduced by LRP to phosphorylated Disheveled (Dsh), which in turn block the activity of glycogen synthase kinase-3β (GSK-3β), a key EMT suppressor that mediates the ubiquitination of Snail and the degradative phosphorylation of β-catenin (Doble and Woodgett, 2007; Heuberger and Birchmeier, 2010; Lam and Gottardi, 2011; Gonzalez and Medici, 2014).

The receptor tyrosine kinases (RTKs) and their downstream effectors are also pivotal in EMT. There are numerous growth factors (GFs), including epidermal GF (EGF), fibroblast GF (FGF), platelet-derived GF (PDGF), and insulin-like GF (IGF), which serve as RTK ligands (Jechlinger et al., 2006; Cevenini et al., 2018; Shu et al., 2018; Yamaoka et al., 2018). Binding of GFs with RTKs and the ensuing tyrosine autophosphorylation of RTKs can result in binding of GTP to Ras and the recruitment of Raf, activating mitogen-activated protein kinase (MAPK)/extracellular signal-regulated protein kinase (ERK) kinase (MEK), which further phosphorylates ERKs. Ligand-bound RTKs also activate phosphoinositide 3-kinase (PI3K), which activates Akt *via* PIP_3_ converted from PIP_2_. Activation of Akt and ERK leads to the inactivating phosphorylation of GSK-3β, which promotes EMT (Oloumi et al., 2004; Zhou et al., 2004; Ding et al., 2005; Doble and Woodgett, 2007; Lemmon and Schlessinger, 2010; Tripathi and Garg, 2018). Akt activation also contributes to signal transduction through the NF-κB/Snail pathway (Julien et al., 2007).

The Janus kinase/signal transducers and activators of transcription-3 (JAK/STAT3) pathway, which were initially defined as the inflammation response-related pathway, also actively participate in EMT. Interleukin-6 (IL-6) activates JAK after binding to IL-6 receptor, which subsequently facilitates the phosphorylation and the following dimerization of STAT3. STAT3 homodimer then translocates into the nucleus and regulate the transcription (Yan et al., 2018). The IL-6/JAK/STAT3 pathway can effectively upregulate pro-EMT TFs, including Snail, Slug, and Twist and modify the expression of EMT-related genes (Colomiere et al., 2009; Sullivan et al., 2009; Yadav et al., 2011; Chen et al., 2015). In addition, interleukin-8 (IL-8) can also induce similar JAK/STAT3-dependent responses through interactions with IL-8 receptor (Fernando et al., 2011; Fu et al., 2015).

The sonic hedgehog (Shh) signaling pathway consisting of Shh, protein patch homolog (PTCH), smoothend (SMO), and glima-associated oncogene-1/2/3 (Gli1/2/3) mediates embryonic development. Recently, the importance of this pathway in cancer and EMT has also been revealed (McCubrey et al., 2016; Zou et al., 2017). Upon binding to the transmembrane G protein-coupled receptor PTCH, secretive Shh releases the repressed SMO-bound PTCH, which further induces the transcriptional activity of Glis, promoting the transcription of Snail, Slug, and other EMT-related gene targets (Li et al., 2006; Pain et al., 2014; Flemban and Qualtrough, 2015; Zhang et al., 2016a).

Novel studies have described the participation of Notch signaling, an original paracrine pathway, in angiogenesis, tumor progression, cell proliferation, and EMT induction. The initiation of Notch relies on the trans-interactions between Notch ligands (Jag1, Jag2, Delta1, Delta3, and Delta4) at cell surface of signaling-sending cells and extracellular domains of noncovalent heterodimeric transmembrane Notch receptors (Notch1, Notch2, Notch3, and Notch4) on the surface of signaling-receiving cells. An initial cleavage at the extracellular site of the notch receptor mediated by a disintegrin and metalloproteinase (ADAM) follows the activation of Notch receptor (Meurette and Mehlen, 2018; Chatterjee and Sil, 2019). After the second intracellular cleavage mediated by γ-secretase, Notch intracellular domain (NICD) with activated transcriptional activity is released and then translocates into the nucleus, mediating the transcriptional upregulation of hairy and enhancer of split (HES) family genes and the HES related with YRPW motif (HEY) family genes in cooperation with co-regulators. These effectors further induce the corresponding downstream transcriptional modification, such as the induction of Snail and Slug (Thiery et al., 2009; Vinson et al., 2016; Leonetti et al., 2019).

**Figure 1:**
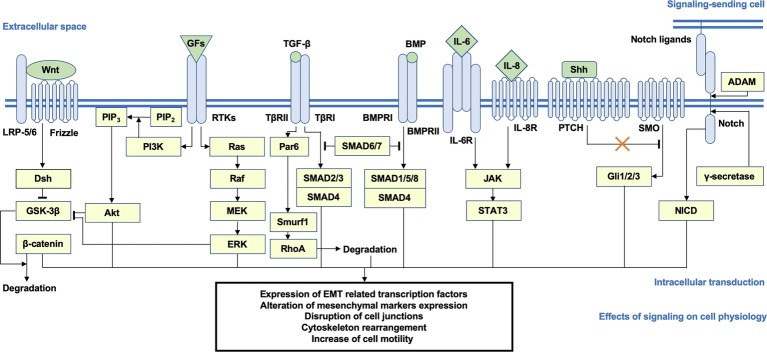
Brief schema of the putative signaling transduction mechanisms underlying EMT. Activation of the Wnt/β-catenin, PI3K/Akt, Ras/ERK, TGF-β/SMAD2/3, BMP/SMAD1/5/8, JAK/STAT3, Shh, and Notch pathways is highly correlated with EMT. After ligand-receptor binding, intracellular secondary messengers are activated and initiated downstream transduction, which generally induce the nuclear translocation of signaling-specific TFs and the transcriptional regulation of EMT-related genes, such as CDH1 and CDH2, EMT TFs, and mesenchymal markers, accompanied by a series of alterations on cellular physiological or pathological activities (e.g., disjunction of adherin junctions, cytoskeleton remodeling, and increase of cellular motility). Arrows represent the molecular interactions in which downstream messengers are activated; T shape arrows represent inhibitive molecular interactions. EMT, epithelial-mesenchymal transition; PI3K, phosphoinositide 3-kinase; ERK, extracellular signal-regulated protein kinase; TGF-β, transforming growth factor β; JAK, Janus kinase; Shh, sonic hedgehog; TFs, transcription factors.

A specific consequence of the multiple signal transduction networks reviewed above is the increase of cell motility. To drive the process of migration and invasion, the subtle remodeling of cytoskeleton dynamics accompanied by the tuning of epithelial adhesion, which is directly regulated by focal adhesion kinase (FAK), Rho family GTPase, and Rho-associated protein kinase (ROCK) signaling, is highly activated, leading to actin polymerization, stress fiber assembly, lamellipodia and filopodia formation, and the creeping-like motions of the cells (Cavallaro and Christofori, 2001; Yilmaz and Christofori, 2009; Jiang et al., 2017; Jansen et al., 2018). A schematic overview of the pro-EMT pathways and key messengers is shown in Figure 1.

## Potential Mechanisms of PM_2.5_-Induced Epithelial-Mesenchymal Transition

### Potential Reactive Oxygen Species-Dependent Mechanisms in PM_2.5_-Induced Epithelial-Mesenchymal Transition

ROS are physiologically produced as byproducts of biological reactions in the cytoplasm, mitochondria, and peroxisomes catalyzed by cytochrome P450 and NADPH oxidase (NOX). ROS have primary functions to actively defend against invading pathogens (Bardaweel et al., 2018; Lee et al., 2018). However, excessive cytoplasmic retention of ROS generally results in cellular oxidative stress, which can alter intracellular signaling, DNA damage, and apoptosis (Danielsen et al., 2009; Deng et al., 2014; Vattanasit et al., 2014; Cho et al., 2018). Intracellular ROS can be generated by cellular exposure to external contaminants, including the main components of PM_2.5_, such as black carbon, redox-active metals (e.g., Fe, Cu, Ni, Zn, Cr, As, and Mn), and PAHs (e.g., naphthalene, benzo-*a*-pyrene, and anthracene) (Shuster-Meiseles et al., 2016; Niranjan and Thakur, 2017; Sun et al., 2018). PM_2.5_ particles have also been characterized as strong inducers of ROS, as they directly release ROS, catalyze redox reactions, or participate in redox cycles as substrates (Squadrito et al., 2001; Knaapen et al., 2004; Risom et al., 2005; Valavanidis et al., 2013; Cho et al., 2018; Jin et al., 2018).

As ROS has been well characterized as one of the fundamental intermediaries of the PM_2.5_-induced response and injury, and evidence verifying PM_2.5_-induced EMT *via* ROS *in vivo* and *in vitro* has been emerging, this section will be mainly focused on the potential pathogenic role of PM_2.5_-derived ROS in EMT (Thevenot et al., 2013; Yan et al., 2016; Yi et al., 2017; Palleschi et al., 2018; Li et al., 2018b). Some essential information including the sources and the concentrations of PM_2.5_ utilized, as well as the antioxidant used for treatment and the effective concentrations, is summarized in Table 2.

**Table 2:** Summary table about exposure assays of some studies reviewed in section “Potential Reactive Oxygen Species-Dependent Mechanisms in PM_2.5_-Induced Epithelial-Mesenchymal Transition.”

Source of PM_2.5_	Dose of PM_2.5_ exposure	Duration of exposure	Cell type	Antioxidant	Dose of antioxidant	Reference
Collected at Atlanta, USA, from March 1, 2004 to June 30, 2004 using Teflon filter	0.1, 1, 10 μg/cm^2^	5 days	Rat alveolar type II epithelial cell line (RLE-6TN cells)	*N*-Acetyl cysteine (NAC)	5 μmol/L	Dysart et al. (2014)
Collected at Beijing, China, from January 19, 2015 to January 21, 2015 using 90-mm Emfab filter	1, 5, 30 μg/cm^2^	Single exposure: 1 day Repeated exposure: 7 days	Human bronchial epithelial cell line (BEAS-2B cells)	NAC	100 μmol/L	Tripathi et al. (2018)
Collected at Changchun, China, from November 2015 to March 2016 using quartz filter	25 μg/cm^2^	1, 6, 12, 24 h	Human bronchial epithelial cell line (BEAS-2B cells)	NAC	5 μmol/L	Song et al. (2017)
Ni ions were bought from Sigma, USA	Ni^2+^: 0.4 μmol/L	24 h	Human lung cancer cell line (A549 cells) Human lung fibroblast cell line (MRC-5 cells)	NAC	5 mmol/L	Yang et al. (2018)
Collected at Guangzhou, China, using quartz filter	20, 50, 100 μg/ml	24 h	Human corneal epithelial cell line (HCEC cells)	NAC	1 mg/ml	Cui et al. (2018)
Bought from National Institute of Standards and Technology (NIST), USA (the product label: SRM 2786)	2.5, 5, 10 μg/ml	24 h	Human hepatic stellate cell line (LX-2 cells) Human primary hepatic stellate cells (HSCs)	NAC	1 mmol/L	Qiu et al. (2019)
Collected at Beijing, China, from January 2009 to June 2009 using nitrocellulose filter	8, 16, 32, 64 μg/cm^2^	12, 24, 48 h	Human lung cancer cell line (A549 cells)	NAC	5 mmol/L	Deng et al. (2017)

#### PM_2.5_-Reactive Oxygen Species Participates in SMAD-Dependent Epithelial-Mesenchymal Transition

TGF-β has been shown to involve ROS formation *via* NOX4, whereas ROS also stimulate the epithelial excretion of TGF-β (Latella, 2018). ROS can also convert latent TGF-β to its active form by oxidizing the latency-associated peptide (LAP) (Liu and Gaston Pravia, 2010; Montorfano et al., 2014; Jaffer et al., 2015). Excessive ROS derived from protein oxidation can induce TβRI/II expression and further initiate TGF-β/SMAD signaling, which induces EMT in hepatocytes *in vivo* and *in vitro* (Sun et al., 2017). These studies indicate that ROS can activate TGF-β/SMAD signaling at different points along the pathway, including ligand-receptor interactions and intracellular signaling.

An *in vivo* study demonstrated that long-term PM_2.5_ exposure induced increased TGF-β1 expression, SMAD2/3 phosphorylation, and collagen accumulation in mice lungs, indicating potential links among PM_2.5_ exposure, the TGF-β1/SMAD2/3 pathway, and pro-fibrotic EMT (Gu et al., 2017). Another study supported this pathogenesis in mice model: Maternal exposure to PM_2.5_ could also result in significant oxidative stress, activation of TGF-β/SMAD3 signaling, and EMT in fetal mice lung, which contributed to postnatal pulmonary dysfunction (Tang et al., 2017). In addition, exposure of mice to PM_2.5_ for 4 weeks not only elicited SMAD3 phosphorylation and cardiac fibrosis but also induced excessive ROS accumulation and NOX4 expression in heart tissues, indicating that the potential occurrence of PM_2.5_-induced EMT or endothelial-mesenchymal transition (EndMT) might be ROS dependent (Qin et al., 2018). Our previous *in vitro* study validated this mechanism: The release of TGF-β1, the activation of the TGF-β1/SMAD3 pathway, and the altered expression of hallmark EMT proteins in human bronchial epithelial cells were observed after 30-passage low-dose PM_2.5_ exposure (Xu et al., 2019a). In another study, the exposure of alveolar epithelial cells to PM_2.5_ not only induced the activation of latent TGF-β but also increased cell contractility and elongated cellular morphology in a dose-dependent manner. These effects could be reversed by NAC treatment, demonstrating the role of ROS in this pathogenic event (Dysart et al., 2014). In addition, increased TGF-β1 mRNA expression was observed in bronchial epithelial cells after 7 days’ consecutive exposure to PM_2.5_, and treatment with the antioxidant NAC could also effectively offset the toxicity of PM_2.5_ exposure (Tripathi et al., 2018). However, the detailed EMT transcription patterns induced by PM_2.5_-induced ROS through the SMAD2/3-dependent pathway remain unknown.

In summary, these data demonstrate that PM_2.5_ might induce EMT *via* ROS in a TGF-β/SMAD2/3 signaling-dependent manner. However, additional research is needed to further establish a firm causal link.

#### PM_2.5_-Reactive Oxygen Species Might Induce Epithelial-Mesenchymal Transition *via* NF-κB Cascades

The activation of NF-κB can be evoked by ROS. NF-κB is activated as the response of cellular oxidative stress, responsible for inflammation, apoptosis, and differentiation. Moreover, the activation of NF-κB by ROS is also correlated with Snail upregulation and EMT phenotype transformation in epithelial cells (Valavanidis et al., 2013; Cichon and Radisky, 2014). Different antioxidant treatments targeting ROS in human non-small cell lung carcinoma cells, pancreatic cancer cells, and hepatocellular carcinomatous cells all effectively inhibit NF-κB activation, which further represses matrix metalloproteinase (MMP) expression together with cellular migration and invasion (Lee and Lim, 2011; Chao et al., 2017; Huang and Xin, 2018). In addition, IL-6 and IL-8, which are transcriptionally upregulated by ROS-activated NF-κB, also exert pro-EMT abilities *via* downstream JAK/STAT3 signaling (Gupta et al., 2012). In gastric cancer cells, even the regulatory effects of IL-6/JAK/STAT3 on expression of pro-EMT TFs and EMT markers relied on NOX4, indicating the potential comprehensive role of ROS in the transduction of NF-κB/IL-6 signaling (Gao et al., 2017). NF-κB is also able to induce TNF-α expression in response to ROS stimulation, and activated TNF-α receptors in turn upregulate NF-κB transcription with further Snail expression, implying positive feedback loops underlying ROS-induced EMT *via* NF-κB (Dong et al., 2007).

A novel publication confirmed the positive role of NF-κB in PM_2.5_-induced EMT. Organic extracts of PM_2.5_ enhanced the binding of NF-κB with the promoter of long non-coding RNA (lncRNA) MALAT1, and MALAT1 relieved the expression silencing of ZEB1 by sponging miR-204 targeting ZEB1 mRNA, resulting in the mesenchymal phenotype transition of pulmonary epithelial cells (Luo et al., 2019). Another novel study showed the correlation between PM2.5, ROS, NF-κB, and pro-fibrotic EMT in vivo. The oxidative stress within the rat lungs exacerbated during PM2.5 exposure but disappeared after the termination of exposure, and the upregulation of RelA/p65, deteriorated pulmonary fibrosis, and EMT in rat lungs were observed in the post-exposure phase, indicating that PM2.5 might lead to pulmonary fibrosis by oxidative stress-initiated NF-κB/inflammation/EMT pathway (Sun et al., 2019). Moreover, a previous study demonstrated that NF-κB activation following PM_2.5_ exposure can be ROS dependent: The inhibition of ROS effectively abrogated the sustained NF-κB activation in BEAS-2B bronchial epithelial cells induced by PM_2.5_ exposure (Song et al., 2017). Mice exposed to ultrafine carbon black particles showed oxidant-dependent NF-κB activation and increased expression of NF-κB responsive genes including TNF-α and IL-6 in lung tissues (Shukla et al., 2000). Silica particles, a kind of well-described pro-fibrotic agent, were able to induce a similar pathological response in silicosis murine models, whereas non-fibrogenic particles failed to induce this effect. These results implied that ROS-derived NF-κB and its downstream inflammatory mediators actively participate in fibrogenesis, in which EMT might act as a major participant (Hubbard et al., 2002). Additionally, an *in vitro* study showed increased IL-6 level and downstream JAK2/STAT3 signaling activation concomitant with the emergence of oxidative stress in PM_2.5_-treated alveolar cancer cells, whereas a similar pattern was also observed in another *in vitro* study, in which ROS accumulation, IL-6/IL-8 release, and JAK2/STAT3 signaling pathway activation were observed to be mediated by cytochrome P450, family 1, member A1/B1 (CYP1A1/B1) after PM_2.5_ exposure to human bronchial epithelial cells (Yuan et al., 2019). This evidence partly substantiated the hypothesis that PM_2.5_ might induce EMT *via* ROS/NF-κB/IL-6/8/JAK/STAT3 axis (Xu et al., 2015). Although the NF-κB activation and following release of IL-6, IL-8, TNF-α, or mitochondrial dysfunction in epithelial tissues induced by PM_2.5_-derived ROS have been validated to be significant in lung inflammation and cell death, the in-depth regulatory role of this shared pathway in EMT is largely unknown (Bourgeois and Owens, 2014; Leclercq et al., 2018; Liu et al., 2018; Wang et al., 2018; Xin et al., 2018). Currently, the link between cigarette smoke extract (CSE) and pulmonary inflammation together with EMT in airway epithelia has been preliminarily verified. CSE-induced inflammatory responses in human bronchial cells include NF-κB activation and further increased IL-6 transcription, which promote phenotype transformation *via* downregulation of miR-200-c, a proven anti-EMT micro-RNA (miRNA) family member. Hence, ROS in conjunction with NF-κB might lie at the intersection of PM_2.5_-derived inflammation and EMT, although the exact mechanisms remain to be elucidated (Zhao et al., 2013). Controversy on this issue also existed. Fe_3_O_4_, one of the major anthropogenic components in PM, could result in a delayed NF-κB response in A549 neoplastic pulmonary epithelial cells as a result of the decrease in IκB degradation induced by excessive ROS (Konczol et al., 2011).

In summary, whether PM_2.5_-intracellular ROS induced NF-κB phosphorylation could initiate EMT is still obscure, as the small amount of peripheral proof is too weak to conclusively establish this stand.

#### PM_2.5_, Reactive Oxygen Species, and Receptor Tyrosine Kinase Signaling

ROS are strong initiators of pro-EMT PI3K/Akt and Ras/ERK signaling. ROS have been shown to directly induce the phosphorylation of EGF and PDGF receptors in the absence of ligand and to enhance VEGF-induced receptor phosphorylation *via* oxidative repression of the intracellular inhibitors protein tyrosine phosphatase-1B (PTP1B) and density-enhanced phosphatase-1 (DEP1) (Lei and Kazlauskas, 2009; Oshikawa et al., 2010; Leon-Buitimea et al., 2012). The ROS-induced conversion of PIP_2_ to PIP_3_ also initiates Akt signaling events (Zhang et al., 2016b). Additionally, glutathione peroxidase inactivation-induced ROS accumulation resulted in the acquisition of mesenchymal characteristics of pancreatic cancer cells by the Akt/GSK-3β/Snail regulation (Meng et al., 2018). Likewise, the inhibition of GSK-3β, which increases Snail transcriptional activity and an EMT-like phenotype following ROS-stimulated ERK activation instead of Akt, was observed in *Helicobacter pylori*-treated gastric cancer cells (Ngo et al., 2016). Besides, the definitive role of ROS in activating ERK to promote the invasion of vascular smooth muscle cells and keratinocytes has also been unfolded. ROS-mediated ERK phosphorylation is also a significant alternative pathway for TGF-β-induced EMT in renal tubular epithelial cells (Rhyu et al., 2005; Wu, 2006).

PM_2.5_ has been extensively regarded to stimulate the ERK or Akt pathway. PM_2.5_ could induce MMP-13 expression, invasion, and migration of hepatocellular carcinoma cells *via* Akt (Zhang et al., 2017a). The increased secretion of EGF by human alveolar epithelial cells after PM_2.5_ exposure and the further initiation of intracellular EGFR/ERK/NF-κB have also been observed (Jeong et al., 2017). In addition, a microfluidic system-based study on PM_2.5_-treated bronchial epithelial cells showed significant activation of PI3K/Akt pathway, FGF/FGFR/MAPK/VEGF signaling, and the JAK/STAT pathway, which led to cell proliferation and apoptosis evasion. And increased intracellular and mitochondrial ROS levels implicated that PM_2.5_-derived ROS could be correlated with the activation of these signaling series (Zheng et al., 2017).

In addition to ERK, other members of the MAPK family have been shown to participate in PM_2.5_ responses. The coordination of p38 MAPK with proliferating cell nuclear antigen (PCNA) was responsible for PM_2.5_-induced vascular smooth muscle cell proliferation, and the positive role of ROS was also implied by upregulation of superoxide dismutase (SOD), a cellular oxidative stress biomarker (Wan et al., 2018). Nevertheless, current research on the toxicology of PM_2.5_-ROS also primarily focuses on the linkage between Akt or MAPK activation and inflammation or apoptosis, suggesting a double-edged role of PM_2.5_-ROS that could either induce cell death or enhance cell viability (An et al., 2018; Li et al., 2018a). Abundant in PM_2.5_, Ni^2+^ was shown to be responsible for cellular oxidative stress and the phenotype transformation of alveolar epithelial cells, and ROS/Akt-dependent MMP-9 and COL1 induction. Similarly, Ni^2+^/ROS/Akt also led to the inflammation of pulmonary epithelia and mice lung tissues (Yang et al., 2018). Hence, there is also crosstalk between PM_2.5_-ROS-induced inflammation and EMT *via* RTK-related cascades. However, further research is needed to clarify the interplay of PM_2.5_, ROS, Akt, and ERK with EMT.

#### PM_2.5_-Derived Reactive Oxygen Species: The Double-Edged Sword on Cytoskeleton Remodeling

Increase of cell invasion/migration is the characteristic of EMT regulated by subtle cytoskeleton remodeling and shifting in adhesion. TGF-β-induced intracellular ROS *via* NOX4 accelerates the polymerization of globular actin (G-actin) to fibrous actin (F-actin) in human umbilical vein endothelial cells, which is essential for lamellipodia and filopodia formation (Hu et al., 2005; Lamouille et al., 2014). ROS generated by NOX1 was also able to induce the spindle-like cellular morphology, EMT, and differentiation of human gingival epithelial cells *via* the post-transcriptional accumulation of CK18, a kind of pro-EMT intermediate filament (Sattayakhom et al., 2014). And ROS/ErbB2 signaling was also reported to be responsible for the aggressiveness of breast epithelial cells by phosphorylating FAK and activating RhoC (Wang et al., 2017). In another study, although mere H_2_O_2_ treatment was not sufficient to induce complete EMT in retinal pigment epithelial cells due to the transiency of the stimulation, ROS could also initiate Rho-GTP binding, resulting in stress fiber formation (Inumaru et al., 2009).

PM_2.5_ has been verified as the modulator of cytoskeleton inducing incomplete contact between adjacent human bronchial epithelial cells, in which strong oxidative stress was also observed (Longhin et al., 2016). Besides, long-term chronic PM_2.5_ exposure to human bronchial epithelial cells could enhance RhoA-GTP binding and stress fiber formation, although no increase of cellular proliferation was observed (Dornhof et al., 2017). In another research, however, PM_2.5_ that stimulated ROS generation in corneal epithelial cells failed to promote EMT *via* cytoskeletal regulation. On the contrary, PM_2.5_ inhibited FAK and paxillin phosphorylation, which further repressed RhoA activity and epithelial cell migration. Now that these inhibitory processes could be reversed with NAC treatment, the discrepant roles of PM_2.5_-derived ROS in cytoskeleton regulation have been identified (Cui et al., 2018). In summary, the lack of comprehensive data leaves the role of PM_2.5_ in cytoskeleton modulation shadowy.

#### Something New: PM_2.5_-Reactive Oxygen Species-Induced Autophagy Participates in Epithelial-Mesenchymal Transition

Autophagy is a cellular degradation process that is activated in response to environmental stressors such as GF depletion and hypoxia (Yang et al., 2015; Marcucci et al., 2017). Recent evidence has linked autophagy and EMT through shared signaling pathways and functional causality in cancer progression and metastasis (Lv et al., 2012; Li et al., 2013; Catalano et al., 2015; Gugnoni et al., 2016; Zhang et al., 2017b). A recent study verified that PM_2.5_ participated in liver fibrosis *via* ROS-mediated mitophagy. Hepatic stellate cells, a principal liver cell type with EMT plasticity, displayed enhanced α-SMA and COL1 expression after PM_2.5_ treatment. These processes were mediated by ROS-induced PTEN-induced kinase 1 (PINK1) and Parkin, which signified mitophagy (Choi et al., 2009; Qiu et al., 2019). In lung cancer cells, PM_2.5_-induced cell migration and invasion depended on autophagy, which was initially induced by increased intracellular ROS and its downstream effector lncRNA, loc146880 (Deng et al., 2017). Another study revealed an alternative mechanism underlying PM_2.5_-autophagy-EMT in lung cancer cells, in which upregulated lncRNA LCPAT1 served as a messenger that mediated autophagy and cell migration by interacting with RCC2 after PM_2.5_ exposure (Lin et al., 2018). This evidence not only validates the correlation between autophagy and PM_2.5_-induced epithelia phenotype transformation but also provides new insight into the epigenetic toxicology of PM_2.5_.

### Other Molecular Events Underlying PM_2.5_-Induced Epithelial-Mesenchymal Transition

Information about exposure assays in this section is selectively summarized in Table 3.

**Table 3:** Summary table about exposure assays of some studies reviewed in section “Other Molecular Events Underlying PM_2.5_-Induced Epithelial-Mesenchymal Transition.”

Source of PM_2.5_ for treatment	Dose of PM_2.5_ exposure	Duration of exposure	Cell type	Reference
***In vitro* assays**				
Collected at Beijing, China, from December 2016 to February 2017 using glass fiber filter	25, 50, 100 μg/ml	Chronic exposure for 5 passages	Human lung cancer cell line (A549 cells) Human bronchial epithelial cell line (BEAS-2B cells)	Wang et al. (2019)
Collected at Shenyang, China, in winter using nitrocellulose filter	5, 10, 20 μg/cm^2^	72 h	Human lung cancer cell line (A549 cells) Human lung cancer cell line (H292 cells)	Yang et al. (2017)
Collected at Shanghai, China, from November 2017 to June 2018 using glass fiber filter	50 μg/mL	Chronic exposure for 30 passages	Human bronchial epithelial cell line (BEAS-2B cells)	Xu et al. (2019b)
Bought from NIST, USA (the product label: SRM 1648a)	100, 500 μg/ml	Chronic exposure for 30 passages	Human bronchial epithelial cell line (HBE cells)	Fu et al. (2019)
Collected at Nanjing and Shanghai, China, in Autumn 2014 using quartz filter	Organic extract of PM_2.5_: 5 μg/ml	48 h	Human lung cancer cell line (A549 cells) Human bronchial epithelial cell line (BEAS-2B cells)	Huang et al. (2017)
Obtained from a biomass power plant	100 μg/ml	5 weeks	Human bronchial epithelial cell line (BEAS-2B cells)	Hesselbach et al. (2017)
Collected at Shanghai, China in four seasons using glass fiber filter	20, 40, 60, 80, 100 μg/ml	24 h	Human bronchial epithelial cell line (BEAS-2B cells)	Zheng et al. (2017)
**Source of PM_2.5_ for treatment**	**Dose of PM_2.5_ exposure**	**Method and duration of exposure**	**Animal**	**Reference**
***In vivo* assays**				
Collected at Beijing, China, from December 2016 to February 2017 using glass fiber filter	2.5, 10, 20 mg/kg Diluted in saline	Intratracheal instillation Once/3 days, lasted for 90 days	8-week-old male BALB/c mice	(Wang et al., 2019)
Bought from NIST, USA (the product label: SRM 1648a)	0.4 mg/m^3^	Dynamic inhalation exposure using the exposure chambers and aerosol generator Lasted for 21 days	8-week-old C57BL/6 mice	(Fu et al., 2019)

#### Participation of Novel Signaling and Molecules in PM_2.5_-Induced Epithelial-Mesenchymal Transition

PM_2.5_ can also activate EMT *via* diverse signaling pathways, including HMGB1-receptor for advanced glycation end-products (RAGE), Shh, Wnt/β-catenin, and Notch pathway together with SMAD1 signaling, in which the role of ROS has not been elucidated. HMGB1 and its cognate receptor RAGE were both induced in human bronchial epithelial cells and in the murine airway in response to PM_2.5_ exposure. HMGB1 binding further activated TGF-β1 and PDGF release, providing strong evidence that HMGB1 is a mediator of PM_2.5_-induced EMT (Zou et al., 2018). Increased Shh expression activated downstream Shh/Gli1 signal transduction and further enhanced Snail expression as well as cell migration after low dose PM_2.5_ exposure to human bronchial smooth muscle cells (Ye et al., 2018). Moreover, the enhanced expression of Notch1 together with downstream TFs Hes1, Snail, Slug and biomarkers of EMT was detected in lungs of mice after intratracheal instillation of PM_2.5_ with varied concentrations. The induction of cancer stem cells markers including OCT4 and SOX2 in A549 neoplastic pulmonary epithelial cells following the activation of PM_2.5_/Notch1 pathway further validated the pro-EMT property of PM_2.5_ (Wang et al., 2019). Furthermore, unlike the SMADs-dependent EMT mechanisms stated above, SMAD1 activity could also be indirectly induced by repressing expression of SMAD6/7 after PM_2.5_ treatment on neoplastic pulmonary epithelial cells, resulting in the inhibition of SMAD1 degradation and induction of the spindle-like cellular morphology with cadherin switch (Yang et al., 2017). Interestingly, rather than directly responding to PM_2.5_ exposure, Wnt/β-catenin signaling promoted the proliferation of human alveolar cancer cells and xenograft tumor growth *via* PM_2.5_-induced Wnt3a-enriched exosomes (Xu et al., 2018). Our recent research was focused on exosomal miRNAs of EMT-like pulmonary epithelia after PM_2.5_ exposure. It predicted the abnormal regulation of multiple cancer-related pathways by exosomal miRNAs in a systematic perspective, further emphasizing the significance of diverse interactions between exosomes and signaling pathways in the process of PM_2.5_-induced EMT (Xu et al., 2019b). ATP citrate lyase (ACLY) linking glycolytic and lipidic metabolism has been considered as a novel anticancer target, and recently, it has also been found responsible for PM_2.5_-induced EMT (Granchi, 2018). Increase of intracellular ACLY expression and citrate acid levels synchronized with EMT phenotype transformation of human bronchial epithelial cells after long-term PM_2.5_ exposure, and the knock down of ACLY significantly abrogated the *in vivo* metastasis ability of the cells (Fu et al., 2019).

#### Polyaromatic Hydrocarbons Might Function in PM_2.5_-Induced Epithelial-Mesenchymal Transition Through Aryl Hydrocarbon Receptor

Showing high affinity for a variety of PAHs, ligand-bound aryl hydrocarbon receptor (AHR) is able to interact with Ah receptor nuclear translocator protein (ARNT) and further mediate the toxicities of PAHs by stimulating the expression of target genes such as CYP1A1, which oxidizes PAHs and generally enhances their toxicities (Safe et al., 2013; Noakes, 2015). The AHR-ARNT complex can also promote cell proliferation by binding to the Slug promoter, activating ERK in the absence of external GFs, and enhancing FGF, PDGFA, and PCNA production (Shimba et al., 2002; Diry et al., 2006; Randi et al., 2008; Wang et al., 2009; Safe et al., 2013; John et al., 2014). Because PAHs account for a large portion of the organic constituents in PM_2.5_, their contribution to PM_2.5_-induced EMT might be nonnegligible. The significant *cyp1a1* upregulation following the exposure of HBE cells to diesel exhaust particles (DEPs) or the exposure of BEAS-2B and A549 cells to PM_2.5_ proved the ability of PM to activate AHR signaling (Gualtieri et al., 2011, 2012; Rynning et al., 2018). Likewise, inhibiting AHR or CYP1A1 in BEAS-2B and A549 epithelial cells before PM_2.5_ exposure could effectively rescue PM_2.5_-induced E-cadherin downregulation and N-cadherin upregulation. In this study, lncRNA MALAT1 showed synchronized expression tendency with N-cadherin, indicating that it functions as an effector regulating EMT at downstream of PM_2.5_/AHR signaling, in addition to its role in NF-κB pathway reviewed above (Huang et al., 2017). These studies indicated that PM_2.5_/PAHs/AHR/CYP1A1 signaling significantly participates in EMT initiation, although more exploration is urgently needed.

#### PM_2.5_ Induces Pro-Epithelial-Mesenchymal Transition Epigenetic Regulations

Epigenetic regulations through lncRNAs and miRNAs underlying PM_2.5_-induced EMT have been partly reviewed above. Besides that, miR-16-1-3p directly targeting Twist mRNA could be inhibited by PM_2.5_ exposure, participating in PM_2.5_ exposure-induced EMT of hepatocellular carcinoma cells (Zhang and Li, 2019). Moreover, PM_2.5_ exposure also participates in initiation of EMT of bronchial epithelial cells by altering DNA methylation (Li et al., 2017). After 5 weeks of PM_2.5_ exposure, hypomethylated CpG islands (CpGs) in the genome of bronchial epithelial cells were significantly enriched in genes associated with GTPase activity, extracellular matrix organization, and GF stimuli, whereas hypermethylated CpGs were clustered in genes responsible for cell adhesion and ion transportation (Hesselbach et al., 2017). Another set of DNA methylation analyses on bronchial epithelial cells after PM_2.5_ exposure for 24 h showed comparable results, as Gene Ontology (GO) terms related with regulation on actin filament and adherin junction were significantly enriched in hypomethylated gene list (Shi et al., 2019). Because these annotated genes play dominant roles in EMT, these *in silico* analyses strongly indicated an association between PM_2.5_ exposure and EMT from a systematic perspective.

#### Calcium Might Act as an Initiator of Epithelial-Mesenchymal Transition in PM_2.5_?

Calcium signaling is highly correlated with multiple malignant cancers. Alterations in the expression of Ca^2+^ channels or transporters have been identified in tumor cells, indicating that ectopic Ca^2+^ signal transduction might play a role in EMT (Cui et al., 2017). For example, the activation of transient receptor potential cation channel 7 (TRPM7) and Ca^2+^ influx through TRPM7 indicates the initiation of EMT. Furthermore, EGF-induced TRPM7-Ca^2+^-dependent STAT3 activation significantly upregulated Twist, Snail, and EMT marker expression in breast cancer cells (Davis et al., 2014). Ca^2+^ is also able to elicit the expression of the ATP-binding cassette subfamily C member 3 (ABCC3), a promoter of Twist and EMT in breast cancer cells (Stewart et al., 2015). As Ca^2+^ has been well characterized as one of the most plentiful cations in PM_2.5_, and PM_2.5_ is able to penetrate into the bloodstream or be taken by cells, PM_2.5_ exposure might result in increased Ca^2+^ concentration in microenvironment around the epithelia or cytoplasm, which could further regulate the activity of pro-EMT Ca^2+^ signaling (Huang et al., 2018; Qin et al., 2018; Xie and Zhao, 2018). In our recent study, pulmonary epithelial cells showed EMT-like phenotype after long-term low dose PM2.5 exposure, and a subset of the intracellular differentially expressed genes was significantly related to the cellular response to calcium ion (Xu et al., 2019b). However, although limited *in vitro* studies have reported that PM_2.5_ exposure could raise the intracellular Ca^2+^ concentration of BEAS-2B cells and another non-epithelial cell line, the biological correlation of this event with EMT was yet to be clearly figured out (Zheng et al., 2017; Zhao et al., 2019).

## Conclusions

**Figure 2:**
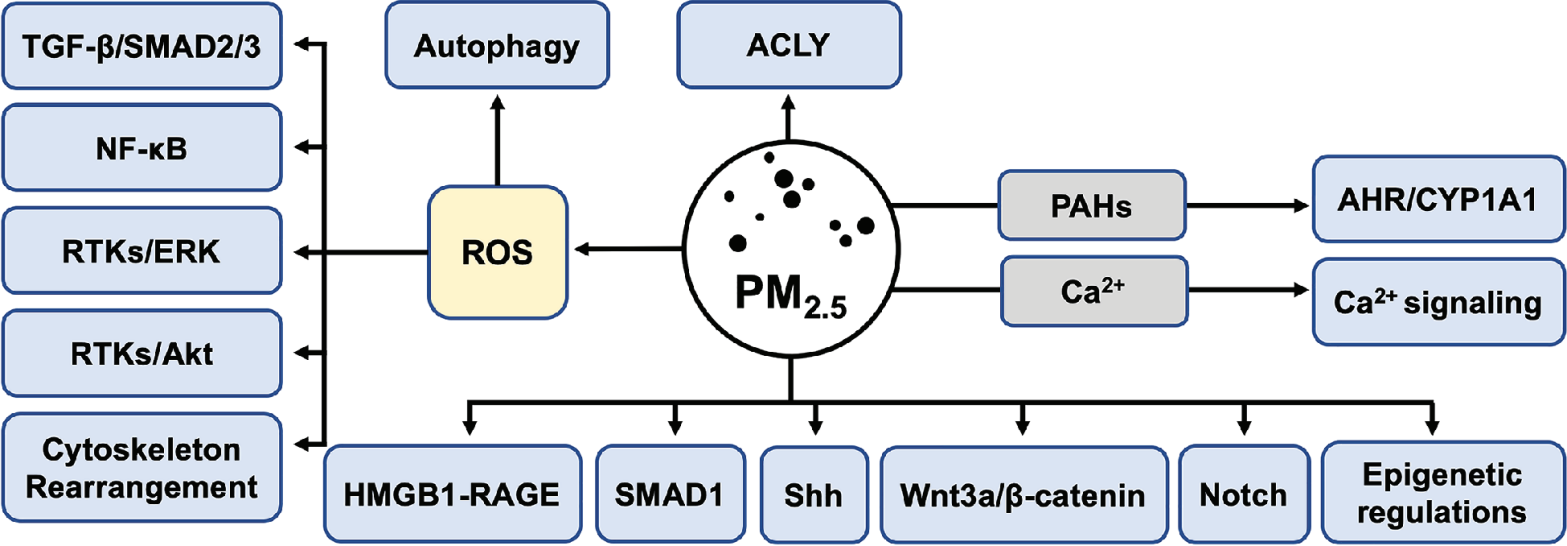
Potential regulatory routes of PM_2.5_ in EMT. PM_2.5_ might function as an EMT initiator by activating TGF-β/SMAD2/3, NF-κB, ERK, Akt, and mediating cytoskeleton rearrangement *via* ROS. In addition to epigenetic mechanisms such as lncRNAs, miRNAs, and DNA methylation regulation, HMGB1-RAGE, Shh, SMAD1, Wnt3a/β-catenin, and Notch pathways have also been originally validated as effector signaling of PM_2.5_ in the process of EMT. ACLY might act as a key metabolic modulator in PM_2.5_-induced EMT. Autophagy following PM_2.5_-ROS can also promote EMT. Specific components within PM_2.5_ might be EMT promoters, as PAHs can initiate EMT *via* AHR/CYP1A1 signaling, and Ca^2+^ ions might function by activating Ca^2+^ signaling. EMT, epithelial-mesenchymal transition; TGF-β, transforming growth factor β; ERK, extracellular signal-regulated protein kinase; ROS, reactive oxygen species; RAGE, receptor for advanced glycation end-products; Shh, sonic hedgehog; ACLY, ATP citrate lyase; PAHs, polyaromatic hydrocarbons; AHR, aryl hydrocarbon receptor.

Accumulating evidence has shown that PM_2.5_ can exert its toxic effect on somatic cells resulting in disorders including carcinogenesis and fibrosis. As EMT endows the epithelia with the characteristics of mesenchymal cells, it has been recognized as one of the major pathogenic mechanisms that promote malignant tumor metastasis and fibrogenesis. We and other researchers have originally identified PM_2.5_ as an initiator of EMT. On the basis of studies investigating PM_2.5_ toxicology, in this review, we describe that PM_2.5_ might induce EMT by intermediary ROS, which induce the secretion of TGF-β, IL-6, IL-8, and TNF-α, as well as the activation of the SMADs, NF-κB, JAK/STAT3, ERK, Akt, and Rho GTPase-dependent cascades. However, only the role of PM_2.5_-derived ROS in EMT induction mediated by TGF-β/SMAD2/3 pathway has been relatively clearly uncovered, whereas other mechanisms are revealed by limited research. Because PM_2.5_-induced inflammation and apoptosis largely share collective key messengers with putative EMT molecular processes, the underlying subtle mechanisms that regulate these cellular events remain to be elucidated. In addition, preliminary evidence has shown a correlation between PM_2.5_-ROS-induced autophagy and EMT, although the interactions at the molecular level remain to be explored. Furthermore, PM_2.5_-induced EMT is also dependent upon HMGB1-RAGE, Shh, Wnt/β-catenin, Notch, SMAD1 signaling, and ACLY enzyme, in which the involvement of ROS remains unclear. It is worth mentioning that epigenetic regulations involving miRNAs, lncRNAs, and DNA methylation might act as transcriptional or posttranscriptional regulators in PM_2.5_-induced EMT. PAHs and Ca^2+^, the primary constituents of PM_2.5_, might specifically induce EMT by AHR and Ca^2+^ signaling, respectively Figure 2.

However, our present understanding of the toxicology of PM_2.5_ is still highly limited, as studies on PM_2.5_ toxicology are confined to a series of factors that might result in ineluctable systematic errors. It is clear that different sampling sites inevitably bring about variance of PM_2.5_ composition, as climate characteristics and social, economic, and cultural features are extremely diverse around the world. This natural diversity might make the results of different studies incomparable. Nevertheless, detailed studies investigating the toxicology of the exact components of PM_2.5_ could be difficult to perform. Second, although sampling protocols have been preliminarily standardized, inconsistencies between the actual operations of researchers uncontrollably exist, which might lead to a reduction in internal authenticity. Besides, the methods of PM_2.5_ treatment, including the selected vehicle solvent and the optimal concentrations to imitate the actual exposure state of humans, also significantly affect the physiology and pathological responses of cultured cells and murine models. According to existing studies, NAC is the widely used antioxidant against PM_2.5_ exposure, which shows strong efficacy of ROS elimination *in vitro*. However, the opposite effect of NAC on lung cancer cells, that is, growth promotion effect by reducing ROS, has been observed (Sayin et al., 2014). Besides, the failure of PANTHER combined therapy consisting of prednisone, azathioprine, and NAC designed for idiopathic pulmonary fibrosis also indicates that precise drug prevention is still far from mature (Idiopathic Pulmonary Fibrosis Clinical Research Network et al., 2012). Therefore, additional in-depth research and consensus are urgently needed. Owing to the relatively limited understanding of the underlying mechanisms of PM_2.5_-induced EMT, we comprehensively reviewed potential molecular events participating in PM_2.5_-induced EMT in various epithelial cell types that might be targeted by PM_2.5_ exposure. As tissue/cell type-specific responses to PM_2.5_ inevitably exist, the definitive role of PM_2.5_ in EMT should be further explored and confirmed.

## Author Contributions

ZX was responsible for literature searching, information aggregation, and manuscript preparation. WD participated in the review and language revision of this manuscript. XD contributed to the concept of this review and participated in the manuscript revision. XD and WD obtained the funding and financially supported the publication of this review. All authors have read and approved the final version of this manuscript.

### Conflict of Interest

The authors declare that the research was conducted in the absence of any commercial or financial relationships that could be construed as a potential conflict of interest.

## Abbreviations

EMT: Epithelial mesenchymal transition
PM_2.5_: Fine particulate matter with an aerodynamic diameter less than 2.5 μm
ROS: Reactive oxygen species
PAHs: Polyaromatic hydrocarbons
ABC: Atmospheric brown cloud
TSP: Total suspended particulates
PM_10_: Inhalable particles with an aerodynamic diameter less than 10 μm
PM_0.1_: Ultrafine particles
α-SMA: α-Smooth muscle actin
TFs: Transcription factors
LEF-1: Lymphoid enhancer-binding factor-1
HMG: High-mobility group protein family
TGF-β: Transforming growth factor β
COL1: Type I collagen
TβRII: The type II TGF-β receptor
TβRI: The type I TGF-β receptor
R-SMADs: Receptor-activated SMAD proteins
BMPR: Bone morphogenetic protein receptor
I-SMADs: Inhibitory SMADs
Wnt: Wingless/Integrated
LRP5/6: LDL receptor-related protein-5/6
Dsh: Disheveled
GSK-3β: Glycogen synthase kinase-3β
RTKs: Receptor tyrosine kinases
GFs: Growth factors
EGF: Epidermal growth factor
FGF: Fibroblast growth factor
PDGF: Platelet-derived growth factor
IGF: Insulin-like growth factor
MEK: MAPK/ERK kinase
ERK: Extracellular signal-regulated protein kinases
PI3K: Phosphoinositide 3-kinase
PKB/Akt: Protein kinase B
JAK: The Janus kinase
STAT3: Signal transducers and activators of transcription-3
IL-6: Interleukin-6
IL-8: Interleukin-8
Shh: The sonic hedgehog
PTCH: Protein patch homolog
SMO: Smoothend
Gli1/2/3: Glima-associated oncogene-1/2/3
ADAM: A disintegrin and metalloproteinase
NICD: Notch intracellular domain
HES: Hairy and enhancer of split family
HEY: The HES related with YRPW motif family
FAK: Focal adhesion kinase
ROCK: Rho-associated protein kinase
NOX: NADPH oxidase
LAP: Latency-associated peptide
EndMT: Endothelial-mesenchymal transition
NAC: *N*-acetyl cysteine
MMP: Matrix metalloproteinase
CYP1A1/B1: Cytochrome P450, family 1, member A1/B1
LncRNA: Long non-coding RNA
CSE: Cigarette smoke extract
MiRNA: Micro-RNA
PTP1B: Protein tyrosine phosphatase-1B
DEP1: Density-enhanced phosphatase-1
MAPK: Mitogen-activated protein kinase
PCNA: Proliferating cell nuclear antigen
SOD: Superoxide dismutase
F-actin: Fibrous actin
G-actin: Globular actin
PINK1: PTEN-induced kinase 1
RAGE: Receptor for advanced glycation end-products
ACLY: ATP citrate lyase
AHR: Aryl hydrocarbon receptor
ARNT: Ah receptor nuclear translocator protein
DEPs: Diesel exhaust particles
CpGs: CpG islands
GO: Gene Ontology
TRPM7: Transient receptor potential cation channel 7
ABCC3: ATP-binding cassette subfamily C member 3.

## References

[ref1] Ahn B. N. Karadeniz F. Kong C. S. Nam K. H. Jang M. S. Seo Y.. (2016). Dioxinodehydroeckol enhances the differentiation of osteoblasts by regulating the expression of Phospho-Smad1/5/8. Mar. Drugs 14:E168. 10.3390/md14090168, PMID: 27649211 PMC5039539

[ref2] An J. Zhou Q. Wu M. Wang L. Zhong Y. Feng J. (2018). Interactions between oxidative stress, autophagy and apoptosis in A549 cells treated with aged black carbon. Toxicol. In Vitro 54, 67–74. 10.1016/j.tiv.2018.09.008 30240709

[ref3] Bardaweel S. K. Gul M. Alzweiri M. Ishaqat A. HA A. L. Bashatwah R. M. (2018). Reactive oxygen species: the dual role in physiological and pathological conditions of the human body. Eurasian J. Med. 50, 193–201. 10.5152/eurasianjmed.2018.17397, PMID: 30515042 PMC6263229

[ref4] Batlle E. Sancho E. Franci C. Dominguez D. Monfar M. Baulida J.. (2000). The transcription factor snail is a repressor of E-cadherin gene expression in epithelial tumour cells. Nat. Cell Biol. 2, 84–89. 10.1038/35000034, PMID: 10655587

[ref5] Belis C. A. Karagulian F. Larsen B. R. Hopke P. K. (2013). Critical review and meta-analysis of ambient particulate matter source apportionment using receptor models in Europe. Atmos. Environ. 69, 94–108. 10.1016/j.atmosenv.2012.11.009

[ref6] Bourgeois B. Owens J. W. (2014). The influence of hurricanes Katrina and Rita on the inflammatory cytokine response and protein expression in A549 cells exposed to PM_2.5_ collected in the Baton Rouge-Port Allen industrial corridor of southeastern Louisiana in 2005. Toxicol. Mech. Methods 24, 220–242. 10.3109/15376516.2014.881945 24401135

[ref7] Burger G. A. Danen E. H. J. Beltman J. B. (2017). Deciphering epithelial-mesenchymal transition regulatory networks in cancer through computational approaches. Front. Oncol. 7:162. 10.3389/fonc.2017.00162, PMID: 28824874 PMC5540937

[ref8] Catalano M. D’Alessandro G. Lepore F. Corazzari M. Caldarola S. Valacca C.. (2015). Autophagy induction impairs migration and invasion by reversing EMT in glioblastoma cells. Mol. Oncol. 9, 1612–1625. 10.1016/j.molonc.2015.04.016, PMID: 26022108 PMC5528793

[ref9] Cavallaro U. Christofori G. (2001). Cell adhesion in tumor invasion and metastasis: loss of the glue is not enough. Biochim. Biophys. Acta 1552, 39–45. 10.1016/s0304-419x(01)00038-5, PMID: 11781114

[ref10] Cevenini A. Orru S. Mancini A. Alfieri A. Buono P. Imperlini E. (2018). Molecular signatures of the insulin-like growth factor 1-mediated epithelial-mesenchymal transition in breast, lung and gastric cancers. Int. J. Mol. Sci. 19:2411. 10.3390/ijms19082411, PMID: 30111747 PMC6122069

[ref11] Chaffer C. L. San Juan B. P. Lim E. Weinberg R. A. (2016). EMT, cell plasticity and metastasis. Cancer Metastasis Rev. 35, 645–654. 10.1007/s10555-016-9648-7, PMID: 27878502

[ref12] Chao W. Deng J. S. Li P. Y. Liang Y. C. Huang G. J. (2017). 3,4-Dihydroxybenzalactone suppresses human non-small cell lung carcinoma cells metastasis via suppression of epithelial to mesenchymal transition, ROS-mediated PI3K/AKT/MAPK/MMP and NFkappaB signaling pathways. Molecules 22:537. 10.3390/molecules22040537, PMID: 28350337 PMC6154291

[ref13] Chatterjee S. Sil P. C. (2019). Targeting the crosstalks of Wnt pathway with hedgehog and notch for cancer therapy. Pharmacol. Res. 142, 251–261. 10.1016/j.phrs.2019.02.027, PMID: 30826456

[ref14] Chen W. Gao Q. Han S. Pan F. Fan W. (2015). The CCL2/CCR2 axis enhances IL-6-induced epithelial-mesenchymal transition by cooperatively activating STAT3-twist signaling. Tumour Biol. 36, 973–981. 10.1007/s13277-014-2717-z 25318604

[ref15] Cheng I. Tseng C. Wu J. Yang J. Conroy S. M. Shariff-Marco S.. (2019). Association between ambient air pollution and breast cancer risk: the multiethnic cohort study. Int. J. Cancer 1–13. 10.1002/ijc.32308, PMID: 30924138 PMC6765455

[ref16] Chi Y. Huang Q. Lin Y. Ye G. Zhu H. Dong S. (2018). Epithelial-mesenchymal transition effect of fine particulate matter from the Yangtze River Delta region in China on human bronchial epithelial cells. J. Environ. Sci. 66, 155–164. 10.1016/j.jes.2017.05.002, PMID: 29628082

[ref17] Cho C. C. Hsieh W. Y. Tsai C. H. Chen C. Y. Chang H. F. Lin C. S. (2018). *In vitro* and *in vivo* experimental studies of PM_2.5_ on disease progression. Int. J. Environ. Res. Public Health 15:E1380. 10.3390/ijerph15071380, PMID: 29966381 PMC6068560

[ref18] Choi E. K. Kim J. G. Kim H. J. Cho J. Y. Jeong H. Park Y.. (2017). Regulation of RhoA GTPase and novel target proteins for ROCK. Small GTPases 3, 1–8. 10.1080/21541248.2017.1364831, PMID: 29199510 PMC7053945

[ref19] Choi S. S. Omenetti A. Witek R. P. Moylan C. A. Syn W. K. Jung Y.. (2009). Hedgehog pathway activation and epithelial-to-mesenchymal transitions during myofibroblastic transformation of rat hepatic cells in culture and cirrhosis. Am. J. Physiol. Gastrointest. Liver Physiol. 297, G1093–G1106. 10.1152/ajpgi.00292.2009, PMID: 19815628 PMC2850083

[ref20] Cichon M. A. Radisky D. C. (2014). ROS-induced epithelial-mesenchymal transition in mammary epithelial cells is mediated by NF-kB-dependent activation of snail. Oncotarget 5, 2827–2838. 10.18632/oncotarget.1940, PMID: 24811539 PMC4058048

[ref21] Colomiere M. Ward A. C. Riley C. Trenerry M. K. Cameron-Smith D. Findlay J.. (2009). Cross talk of signals between EGFR and IL-6R through JAK2/STAT3 mediate epithelial-mesenchymal transition in ovarian carcinomas. Br. J. Cancer 100, 134–144. 10.1038/sj.bjc.6604794, PMID: 19088723 PMC2634691

[ref22] Cui Y. H. Hu Z. X. Gao Z. X. Song X. L. Feng Q. Y. Yang G.. (2018). Airborne particulate matter impairs corneal epithelial cells migration via disturbing FAK/RhoA signaling pathway and cytoskeleton organization. Nanotoxicology 12, 312–324. 10.1080/17435390.2018.1440651, PMID: 29463199

[ref23] Cui C. Merritt R. Fu L. Pan Z. (2017). Targeting calcium signaling in cancer therapy. Acta Pharm. Sin. B 7, 3–17. 10.1016/j.apsb.2016.11.001, PMID: 28119804 PMC5237760

[ref24] Danielsen P. H. Loft S. Kocbach A. Schwarze P. E. Moller P. (2009). Oxidative damage to DNA and repair induced by Norwegian wood smoke particles in human A549 and THP-1 cell lines. Mutat. Res. 674, 116–122. 10.1016/j.mrgentox.2008.10.014 19041418

[ref25] Dave N. Guaita-Esteruelas S. Gutarra S. Frias A. Beltran M. Peiro S.. (2011). Functional cooperation between Snail1 and twist in the regulation of ZEB1 expression during epithelial to mesenchymal transition. J. Biol. Chem. 286, 12024–12032. 10.1074/jbc.M110.168625, PMID: 21317430 PMC3069405

[ref26] Davis F. M. Azimi I. Faville R. A. Peters A. A. Jalink K. Putney J. W. Jr.. (2014). Induction of epithelial-mesenchymal transition (EMT) in breast cancer cells is calcium signal dependent. Oncogene 33, 2307–2316. 10.1038/onc.2013.187, PMID: 23686305 PMC3917976

[ref27] Deng X. Feng N. Zheng M. Ye X. Lin H. Yu X.. (2017). PM_2.5_ exposure-induced autophagy is mediated by lncRNA loc146880 which also promotes the migration and invasion of lung cancer cells. Biochim. Biophys. Acta Gen. Subj. 1861, 112–125. 10.1016/j.bbagen.2016.11.009, PMID: 27836757

[ref28] Deng X. Zhang F. Wang L. Rui W. Long F. Zhao Y.. (2014). Airborne fine particulate matter induces multiple cell death pathways in human lung epithelial cells. Apoptosis 19, 1099–1112. 10.1007/s10495-014-0980-5, PMID: 24722831

[ref29] Ding Q. Xia W. Liu J. C. Yang J. Y. Lee D. F. Xia J.. (2005). Erk associates with and primes GSK-3beta for its inactivation resulting in upregulation of beta-catenin. Mol. Cell 19, 159–170. 10.1016/j.molcel.2005.06.009, PMID: 16039586

[ref30] Diry M. Tomkiewicz C. Koehle C. Coumoul X. Bock K. W. Barouki R.. (2006). Activation of the dioxin/aryl hydrocarbon receptor (AhR) modulates cell plasticity through a JNK-dependent mechanism. Oncogene 25, 5570–5574. 10.1038/sj.onc.1209553, PMID: 16619036

[ref31] Doble B. W. Woodgett J. R. (2007). Role of glycogen synthase kinase-3 in cell fate and epithelial-mesenchymal transitions. Cells Tissues Organs 185, 73–84. 10.1159/000101306, PMID: 17587811

[ref32] Dong R. Wang Q. He X. L. Chu Y. K. Lu J. G. Ma Q. J. (2007). Role of nuclear factor kappa B and reactive oxygen species in the tumor necrosis factor-alpha-induced epithelial-mesenchymal transition of MCF-7 cells. Braz. J. Med. Biol. Res. 40, 1071–1078. 10.1590/S0100-879X2007000800007, PMID: 17665043

[ref33] Dornhof R. Maschowski C. Osipova A. Giere R. Seidl M. Merfort I.. (2017). Stress fibers, autophagy and necrosis by persistent exposure to PM_2.5_ from biomass combustion. PLoS One 12:e0180291. 10.1371/journal.pone.0180291, PMID: 28671960 PMC5495337

[ref34] Dysart M. M. Galvis B. R. Russell A. G. Barker T. H. (2014). Environmental particulate (PM_2.5_) augments stiffness-induced alveolar epithelial cell mechanoactivation of transforming growth factor beta. PLoS One 9:e106821. 10.1371/journal.pone.0106821, PMID: 25226160 PMC4167324

[ref35] Fabregat I. Caballero-Diaz D. (2018). Transforming growth factor-beta-induced cell plasticity in liver fibrosis and hepatocarcinogenesis. Front. Oncol. 8:357. 10.3389/fonc.2018.00357, PMID: 30250825 PMC6139328

[ref36] Falcon-Rodriguez C. I. Osornio-Vargas A. R. Sada-Ovalle I. Segura-Medina P. (2016). Aeroparticles, composition, and lung diseases. Front. Immunol. 7:3. 10.3389/fimmu.2016.00003, PMID: 26834745 PMC4719080

[ref37] Feng S. Gao D. Liao F. Zhou F. Wang X. (2016). The health effects of ambient PM_2.5_ and potential mechanisms. Ecotoxicol. Environ. Saf. 128, 67–74. 10.1016/j.ecoenv.2016.01.030, PMID: 26896893

[ref38] Fernandez I. E. Eickelberg O. (2012). The impact of TGF-beta on lung fibrosis: from targeting to biomarkers. Proc. Am. Thorac. Soc. 9, 111–116. 10.1513/pats.201203-023AW, PMID: 22802283

[ref39] Fernando R. I. Castillo M. D. Litzinger M. Hamilton D. H. Palena C. (2011). IL-8 signaling plays a critical role in the epithelial-mesenchymal transition of human carcinoma cells. Cancer Res. 71, 5296–5306. 10.1158/0008-5472.CAN-11-0156, PMID: 21653678 PMC3148346

[ref40] Flemban A. Qualtrough D. (2015). The potential role of hedgehog signaling in the luminal/basal phenotype of breast epithelia and in breast cancer invasion and metastasis. Cancer 7, 1863–1884. 10.3390/cancers7030866, PMID: 26389956 PMC4586799

[ref41] Fu X. T. Dai Z. Song K. Zhang Z. J. Zhou Z. J. Zhou S. L.. (2015). Macrophage-secreted IL-8 induces epithelial-mesenchymal transition in hepatocellular carcinoma cells by activating the JAK2/STAT3/snail pathway. Int. J. Oncol. 46, 587–596. 10.3892/ijo.2014.2761, PMID: 25405790

[ref42] Fu Y. Lu R. Cui J. Sun H. Yang H. Meng Q.. (2019). Inhibition of ATP citrate lyase (ACLY) protects airway epithelia from PM_2.5_-induced epithelial-mesenchymal transition. Ecotoxicol. Environ. Saf. 167, 309–316. 10.1016/j.ecoenv.2018.10.033, PMID: 30343145

[ref43] Gao X. Sun J. Huang C. Hu X. Jiang N. Lu C. (2017). RNAi-mediated silencing of NOX4 inhibited the invasion of gastric cancer cells through JAK2/STAT3 signaling. Am. J. Transl. Res. 9, 4440–4449. PMID: 29118906 PMC5666053

[ref44] Ghahhari N. M. Babashah S. (2015). Interplay between microRNAs and WNT/beta-catenin signalling pathway regulates epithelial-mesenchymal transition in cancer. Eur. J. Cancer 51, 1638–1649. 10.1016/j.ejca.2015.04.021, PMID: 26025765

[ref45] Ghio A. J. Carraway M. S. Madden M. C. (2012). Composition of air pollution particles and oxidative stress in cells, tissues, and living systems. J. Toxicol. Environ. Health B Crit. Rev. 15, 1–21. 10.1080/10937404.2012.632359 22202227

[ref46] Gilles C. Polette M. Mestdagt M. Nawrocki-Raby B. Ruggeri P. Birembaut P.. (2003). Transactivation of vimentin by beta-catenin in human breast cancer cells. Cancer Res. 63, 2658–2664. PMID: 12750294

[ref47] Gonzalez D. M. Medici D. (2014). Signaling mechanisms of the epithelial-mesenchymal transition. Sci. Signal. 7:re8. 10.1126/scisignal.2005189, PMID: 25249658 PMC4372086

[ref48] Gonzalez-Nunez M. Munoz-Felix J. M. Lopez-Novoa J. M. (2013). The ALK-1/Smad1 pathway in cardiovascular physiopathology. A new target for therapy? Biochim. Biophys. Acta 1832, 1492–1510. 10.1016/j.bbadis.2013.05.016 23707512

[ref49] Granchi C. (2018). ATP citrate lyase (ACLY) inhibitors: an anti-cancer strategy at the crossroads of glucose and lipid metabolism. Eur. J. Med. Chem. 157, 1276–1291. 10.1016/j.ejmech.2018.09.001, PMID: 30195238

[ref50] Gu L. Z. Sun H. Chen J. H. (2017). Histone deacetylases 3 deletion restrains PM_2.5_-induced mice lung injury by regulating NF-kappaB and TGF-beta/Smad2/3 signaling pathways. Biomed. Pharmacother. 85, 756–762. 10.1016/j.biopha.2016.11.094, PMID: 27919737

[ref51] Guaita S. Puig I. Franci C. Garrido M. Dominguez D. Batlle E.. (2002). Snail induction of epithelial to mesenchymal transition in tumor cells is accompanied by MUC1 repression and ZEB1 expression. J. Biol. Chem. 277, 39209–39216. 10.1074/jbc.M206400200, PMID: 12161443

[ref52] Gualtieri M. Longhin E. Mattioli M. Mantecca P. Tinaglia V. Mangano E.. (2012). Gene expression profiling of A549 cells exposed to Milan PM_2.5_. Toxicol. Lett. 209, 136–145. 10.1016/j.toxlet.2011.11.015, PMID: 22178795

[ref53] Gualtieri M. Ovrevik J. Mollerup S. Asare N. Longhin E. Dahlman H. J. (2011). Airborne urban particles (Milan winter-PM_2.5_) cause mitotic arrest and cell death: effects on DNA, mitochondria, AhR binding and spindle organization. Mutat. Res. 713, 18–31. 10.1016/j.mrfmmm.2011.05.011 21645525

[ref54] Gugnoni M. Sancisi V. Manzotti G. Gandolfi G. Ciarrocchi A. (2016). Autophagy and epithelial-mesenchymal transition: an intricate interplay in cancer. Cell Death Dis. 7:e2520. 10.1038/cddis.2016.415, PMID: 27929542 PMC5260980

[ref55] Guo Y. Xiao L. Sun L. Liu F. (2012). Wnt/beta-catenin signaling: a promising new target for fibrosis diseases. Physiol. Res. 61, 337–346. PMID: 22670697 10.33549/physiolres.932289

[ref56] Gupta S. C. Hevia D. Patchva S. Park B. Koh W. Aggarwal B. B. (2012). Upsides and downsides of reactive oxygen species for cancer: the roles of reactive oxygen species in tumorigenesis, prevention, and therapy. Antioxid. Redox Signal. 16, 1295–1322. 10.1089/ars.2011.4414, PMID: 22117137 PMC3324815

[ref57] Hesselbach K. Kim G. J. Flemming S. Haupl T. Bonin M. Dornhof R.. (2017). Disease relevant modifications of the methylome and transcriptome by particulate matter (PM_2.5_) from biomass combustion. Epigenetics 12, 779–792. 10.1080/15592294.2017.1356555, PMID: 28742980 PMC5739103

[ref58] Heuberger J. Birchmeier W. (2010). Interplay of cadherin-mediated cell adhesion and canonical Wnt signaling. Cold Spring Harb. Perspect. Biol. 2:a002915. 10.1101/cshperspect.a002915, PMID: 20182623 PMC2828280

[ref59] Hu T. Ramachandrarao S. P. Siva S. Valancius C. Zhu Y. Mahadev K.. (2005). Reactive oxygen species production via NADPH oxidase mediates TGF-beta-induced cytoskeletal alterations in endothelial cells. Am. J. Physiol. Ren. Physiol. 289, F816–F825. 10.1152/ajprenal.00024.2005, PMID: 16159901 PMC1460011

[ref60] Huang F. Chen Y. G. (2012). Regulation of TGF-beta receptor activity. Cell Biosci. 2:9. 10.1186/2045-3701-2-9, PMID: 22420375 PMC3333473

[ref61] Huang Q. Chi Y. Deng J. Liu Y. Lu Y. Chen J.. (2017). Fine particulate matter 2.5 exerted its toxicological effect by regulating a new layer, long non-coding RNA. Sci. Rep. 7:9392. 10.1038/s41598-017-09818-6, PMID: 28839203 PMC5570922

[ref62] Huang M. Xin W. (2018). Matrine inhibiting pancreatic cells epithelial-mesenchymal transition and invasion through ROS/NF-kappaB/MMPs pathway. Life Sci. 192, 55–61. 10.1016/j.lfs.2017.11.024, PMID: 29155301

[ref63] Huang X. Zhang J. Luo B. Wang L. Tang G. Liu Z.. (2018). Water-soluble ions in PM2.5 during spring haze and dust periods in Chengdu, China: variations, nitrate formation and potential source areas. Environ. Pollut. 243, 1740–1749. 10.1016/j.envpol.2018.09.126, PMID: 30408861

[ref64] Hubbard A. K. Timblin C. R. Shukla A. Rincon M. Mossman B. T. (2002). Activation of NF-kappaB-dependent gene expression by silica in lungs of luciferase reporter mice. Am. J. Phys. Lung Cell. Mol. Phys. 282, L968–L975. 10.1152/ajplung.00327.2001, PMID: 11943661

[ref65] International Agency for Research on Cancer (IARC) (2019). Agents classified by the IARC monographs. Available from: https://monographs.iarc.fr/agents-classified-by-the-iarc/

[ref66] Idiopathic Pulmonary Fibrosis Clinical Research Network Raghu G. Anstrom K. J. King T. E. Jr. Lasky J. A. Martinez F. J. (2012). Prednisone, azathioprine, and N-acetylcysteine for pulmonary fibrosis. N. Engl. J. Med. 366, 1968–1977. 10.1056/NEJMoa1113354, PMID: 22607134 PMC3422642

[ref67] Inumaru J. Nagano O. Takahashi E. Ishimoto T. Nakamura S. Suzuki Y.. (2009). Molecular mechanisms regulating dissociation of cell-cell junction of epithelial cells by oxidative stress. Genes Cells 14, 703–716. 10.1111/j.1365-2443.2009.01303.x, PMID: 19422420

[ref68] Itoh S. ten Dijke P. (2007). Negative regulation of TGF-beta receptor/Smad signal transduction. Curr. Opin. Cell Biol. 19, 176–184. 10.1016/j.ceb.2007.02.015, PMID: 17317136

[ref69] Jaffer O. A. Carter A. B. Sanders P. N. Dibbern M. E. Winters C. J. Murthy S.. (2015). Mitochondrial-targeted antioxidant therapy decreases transforming growth factor-beta-mediated collagen production in a murine asthma model. Am. J. Respir. Cell Mol. Biol. 52, 106–115. 10.1165/rcmb.2013-0519OC, PMID: 24988374 PMC4370251

[ref70] Jansen S. Gosens R. Wieland T. Schmidt M. (2018). Paving the rho in cancer metastasis: rho GTPases and beyond. Pharmacol. Ther. 183, 1–21. 10.1016/j.pharmthera.2017.09.002, PMID: 28911825

[ref71] Jechlinger M. Sommer A. Moriggl R. Seither P. Kraut N. Capodiecci P.. (2006). Autocrine PDGFR signaling promotes mammary cancer metastasis. J. Clin. Invest. 116, 1561–1570. 10.1172/JCI24652, PMID: 16741576 PMC1469776

[ref72] Jeong S. C. Cho Y. Song M. K. Lee E. Ryu J. C. (2017). Epidermal growth factor receptor (EGFR)-MAPK-nuclear factor(NF)-kappaB-IL8: a possible mechanism of particulate matter(PM) 2.5-induced lung toxicity. Environ. Toxicol. 32, 1628–1636. 10.1002/tox.22390, PMID: 28101945

[ref73] Jiang J. Wang K. Chen Y. Chen H. Nice E. C. Huang C. (2017). Redox regulation in tumor cell epithelial-mesenchymal transition: molecular basis and therapeutic strategy. Signal Transduct. Target. Ther. 2:17036. 10.1038/sigtrans.2017.36, PMID: 29263924 PMC5661624

[ref74] Jin X. Xue B. Zhou Q. Su R. Li Z. (2018). Mitochondrial damage mediated by ROS incurs bronchial epithelial cell apoptosis upon ambient PM_2.5_ exposure. J. Toxicol. Sci. 43, 101–111. 10.2131/jts.43.101, PMID: 29479032

[ref75] John K. Lahoti T. S. Wagner K. Hughes J. M. Perdew G. H. (2014). The ah receptor regulates growth factor expression in head and neck squamous cell carcinoma cell lines. Mol. Carcinog. 53, 765–776. 10.1002/mc.22032, PMID: 23625689 PMC4388041

[ref76] Julien S. Puig I. Caretti E. Bonaventure J. Nelles L. van Roy F.. (2007). Activation of NF-kappaB by Akt upregulates snail expression and induces epithelium mesenchyme transition. Oncogene 26, 7445–7456. 10.1038/sj.onc.1210546, PMID: 17563753

[ref77] Kalluri R. (2009). EMT: when epithelial cells decide to become mesenchymal-like cells. J. Clin. Invest. 119, 1417–1419. 10.1172/JCI39675, PMID: 19487817 PMC2689122

[ref78] Kalluri R. Weinberg R. A. (2009). The basics of epithelial-mesenchymal transition. J. Clin. Invest. 119, 1420–1428. 10.1172/JCI39104, PMID: 19487818 PMC2689101

[ref79] Katsuno Y. Lamouille S. Derynck R. (2013). TGF-beta signaling and epithelial-mesenchymal transition in cancer progression. Curr. Opin. Oncol. 25, 76–84. 10.1097/CCO.0b013e32835b6371, PMID: 23197193

[ref80] Kim H. B. Shim J. Y. Park B. Lee Y. J. (2018). Long-term exposure to air pollutants and cancer mortality: a meta-analysis of cohort studies. Int. J. Environ. Res. Public Health 15, 1–15. 10.3390/ijerph15112608, PMID: 30469439 PMC6266691

[ref81] Knaapen A. M. Borm P. J. Albrecht C. Schins R. P. (2004). Inhaled particles and lung cancer. Part A: mechanisms. Int. J. Cancer 109, 799–809. 10.1002/ijc.11708, PMID: 15027112

[ref82] Koganti P. Levy-Cohen G. Blank M. (2018). Smurfs in protein homeostasis, signaling, and cancer. Front. Oncol. 8:295. 10.3389/fonc.2018.00295, PMID: 30116722 PMC6082930

[ref83] Konczol M. Ebeling S. Goldenberg E. Treude F. Gminski R. Giere R.. (2011). Cytotoxicity and genotoxicity of size-fractionated iron oxide (magnetite) in A549 human lung epithelial cells: role of ROS, JNK, and NF-kappaB. Chem. Res. Toxicol. 24, 1460–1475. 10.1021/tx200051s, PMID: 21761924

[ref84] Kourtidis A. Lu R. Pence L. J. Anastasiadis P. Z. (2017). A central role for cadherin signaling in cancer. Exp. Cell Res. 358, 78–85. 10.1016/j.yexcr.2017.04.006, PMID: 28412244 PMC5544584

[ref85] Lam A. P. Gottardi C. J. (2011). Beta-catenin signaling: a novel mediator of fibrosis and potential therapeutic target. Curr. Opin. Rheumatol. 23, 562–567. 10.1097/BOR.0b013e32834b3309, PMID: 21885974 PMC3280691

[ref86] Lambertini E. Franceschetti T. Torreggiani E. Penolazzi L. Pastore A. Pelucchi S.. (2010). SLUG: a new target of lymphoid enhancer factor-1 in human osteoblasts. BMC Mol. Biol. 11:13. 10.1186/1471-2199-11-13, PMID: 20128911 PMC2834684

[ref87] Lamouille S. Xu J. Derynck R. (2014). Molecular mechanisms of epithelial-mesenchymal transition. Nat. Rev. Mol. Cell Biol. 15, 178–196. 10.1038/nrm3758, PMID: 24556840 PMC4240281

[ref88] Latella G. (2018). Redox imbalance in intestinal fibrosis: beware of the TGFbeta-1, ROS, and Nrf2 connection. Dig. Dis. Sci. 63, 312–320. 10.1007/s10620-017-4887-1, PMID: 29273848

[ref89] Leclercq B. Kluza J. Antherieu S. Sotty J. Alleman L. Y. Perdrix E.. (2018). Air pollution-derived PM_2.5_ impairs mitochondrial function in healthy and chronic obstructive pulmonary diseased human bronchial epithelial cells. Environ. Pollut. 243, 1434–1449. 10.1016/j.envpol.2018.09.062, PMID: 30278417

[ref90] Lee B. W. L. Ghode P. Ong D. S. T. (2018). Redox regulation of cell state and fate. Redox Biol. 10.1016/j.redox.2018.11.014, PMID: 30509603 PMC6859564

[ref91] Lee J. Lim K. T. (2011). Inhibitory effect of phytoglycoprotein (38 kDa) on expression of matrix metalloproteinase-9 in 12-O-tetradecanoylphorbol-13-acetate-treated HepG2cells. Naunyn Schmiedeberg's Arch. Pharmacol. 384, 185–196. 10.1007/s00210-011-0663-5, PMID: 21713380

[ref92] Lei H. Kazlauskas A. (2009). Growth factors outside of the platelet-derived growth factor (PDGF) family employ reactive oxygen species/Src family kinases to activate PDGF receptor alpha and thereby promote proliferation and survival of cells. J. Biol. Chem. 284, 6329–6336. 10.1074/jbc.M808426200, PMID: 19126548 PMC2649107

[ref93] Lemmon M. A. Schlessinger J. (2010). Cell signaling by receptor tyrosine kinases. Cell 141, 1117–1134. 10.1016/j.cell.2010.06.011, PMID: 20602996 PMC2914105

[ref94] Leon-Buitimea A. Rodriguez-Fragoso L. Lauer F. T. Bowles H. Thompson T. A. Burchiel S. W. (2012). Ethanol-induced oxidative stress is associated with EGF receptor phosphorylation in MCF-10A cells overexpressing CYP2E1. Toxicol. Lett. 209, 161–165. 10.1016/j.toxlet.2011.12.009, PMID: 22222162 PMC3641856

[ref95] Leonetti A. Facchinetti F. Minari R. Cortellini A. Rolfo C. D. Giovannetti E.. (2019). Notch pathway in small-cell lung cancer: from preclinical evidence to therapeutic challenges. Cell. Oncol. 42, 261–273. 10.1007/s13402-019-00441-3, PMID: 30968324 PMC12994342

[ref96] Li X. Deng W. Nail C. D. Bailey S. K. Kraus M. H. Ruppert J. M.. (2006). Snail induction is an early response to Gli1 that determines the efficiency of epithelial transformation. Oncogene 25, 609–621. 10.1038/sj.onc.1209077, PMID: 16158046 PMC1361531

[ref97] Li J. H. Li W. X. Bai C. X. Song Y. L. (2017). Particulate matter-induced epigenetic changes and lung cancer. Clin. Respir. J. 11, 539–546. 10.1111/crj.12389, PMID: 26403658 PMC7310573

[ref99] Li A. Xia X. Yeh J. Kua H. Liu H. Mishina Y.. (2014). PDGF-AA promotes osteogenic differentiation and migration of mesenchymal stem cell by down-regulating PDGFRalpha and derepressing BMP-Smad1/5/8 signaling. PLoS One 9:e113785. 10.1371/journal.pone.0113785, PMID: 25470749 PMC4254917

[ref100] Li J. Yang B. Zhou Q. Wu Y. Shang D. Guo Y.. (2013). Autophagy promotes hepatocellular carcinoma cell invasion through activation of epithelial-mesenchymal transition. Carcinogenesis 34, 1343–1351. 10.1093/carcin/bgt063, PMID: 23430956

[ref101] Li J. Zhou Q. Yang T. Li Y. Zhang Y. Wang J. (2018a). SGK1 inhibits PM_2.5_-induced apoptosis and oxidative stress in human lung alveolar epithelial A549cells. Biochem. Biophys. Res. Commun. 496, 1291–1295. 10.1016/j.bbrc.2018.02.002 29412164

[ref102] Li R. Zhou R. Zhang J. (2018b). Function of PM_2.5_ in the pathogenesis of lung cancer and chronic airway inflammatory diseases. Oncol. Lett. 15, 7506–7514. 10.3892/ol.2018.8355 29725457 PMC5920433

[ref103] Lin H. Zhang X. Feng N. Wang R. Zhang W. Deng X.. (2018). LncRNA LCPAT1 mediates smoking/ particulate matter 2.5-induced cell autophagy and epithelial-mesenchymal transition in lung cancer cells via RCC2. Cell. Physiol. Biochem. 47, 1244–1258. 10.1159/000490220, PMID: 29913439

[ref104] Liu R. M. Gaston Pravia K. A. (2010). Oxidative stress and glutathione in TGF-beta-mediated fibrogenesis. Free Radic. Biol. Med. 48, 1–15. 10.1016/j.freeradbiomed.2009.09.026, PMID: 19800967 PMC2818240

[ref105] Liu L. Wan C. Zhang W. Guan L. Tian G. Zhang F.. (2018). MiR-146a regulates PM1 -induced inflammation via NF-kappaB signaling pathway in BEAS-2B cells. Environ. Toxicol. 33, 743–751. 10.1002/tox.22561, PMID: 29667303

[ref106] Liu B. Wu S. D. Shen L. J. Zhao T. X. Wei Y. Tang X. L.. (2019). Spermatogenesis dysfunction induced by PM_2.5_ from automobile exhaust via the ROS-mediated MAPK signaling pathway. Ecotoxicol. Environ. Saf. 167, 161–168. 10.1016/j.ecoenv.2018.09.118, PMID: 30326357

[ref107] Longhin E. Capasso L. Battaglia C. Proverbio M. C. Cosentino C. Cifola I.. (2016). Integrative transcriptomic and protein analysis of human bronchial BEAS-2B exposed to seasonal urban particulate matter. Environ. Pollut. 209, 87–98. 10.1016/j.envpol.2015.11.013, PMID: 26647171

[ref108] Lu W. Kang Y. (2019). Epithelial-mesenchymal plasticity in cancer progression and metastasis. Dev. Cell 49, 361–374. 10.1016/j.devcel.2019.04.010, PMID: 31063755 PMC6506183

[ref109] Luo F. Wei H. Guo H. Li Y. Feng Y. Bian Q.. (2019). LncRNA MALAT1, a lncRNA acting via the miR-204/ZEB1 pathway, mediates the EMT induced by organic extract of PM_2.5_ in lung bronchial epithelial cells. Am. J. Phys. Lung Cell. Mol. Phys. 317, L87–L98. 10.1152/ajplung.00073.2019, PMID: 31042084

[ref110] Lv Q. Wang W. Xue J. Hua F. Mu R. Lin H.. (2012). DEDD interacts with PI3KC3 to activate autophagy and attenuate epithelial-mesenchymal transition in human breast cancer. Cancer Res. 72, 3238–3250. 10.1158/0008-5472.CAN-11-3832, PMID: 22719072

[ref111] Marcucci F. Ghezzi P. Rumio C. (2017). The role of autophagy in the cross-talk between epithelial-mesenchymal transitioned tumor cells and cancer stem-like cells. Mol. Cancer 16:3. 10.1186/s12943-016-0573-8, PMID: 28137290 PMC5282816

[ref112] McCormack N. Molloy E. L. O'Dea S. (2013). Bone morphogenetic proteins enhance an epithelial-mesenchymal transition in normal airway epithelial cells during restitution of a disrupted epithelium. Respir. Res. 14:36. 10.1186/1465-9921-14-36, PMID: 23509993 PMC3607850

[ref113] McCubrey J. A. Rakus D. Gizak A. Steelman L. S. Abrams S. L. Lertpiriyapong K. (2016). Effects of mutations in Wnt/beta-catenin, hedgehog, notch and PI3K pathways on GSK-3 activity-diverse effects on cell growth, metabolism and cancer. Biochim. Biophys. Acta 1863, 2942–2976. 10.1016/j.bbamcr.2016.09.004 27612668

[ref114] Meng Q. Shi S. Liang C. Liang D. Hua J. Zhang B.. (2018). Abrogation of glutathione peroxidase-1 drives EMT and chemoresistance in pancreatic cancer by activating ROS-mediated Akt/GSK3beta/snail signaling. Oncogene 37, 5843–5857. 10.1038/s41388-018-0392-z, PMID: 29980787

[ref115] Meurette O. Mehlen P. (2018). Notch signaling in the tumor microenvironment. Cancer Cell 34, 536–548. 10.1016/j.ccell.2018.07.009, PMID: 30146333

[ref116] Min C. Eddy S. F. Sherr D. H. Sonenshein G. E. (2008). NF-kappaB and epithelial to mesenchymal transition of cancer. J. Cell. Biochem. 104, 733–744. 10.1002/jcb.21695, PMID: 18253935

[ref117] Miyazawa K. Shinozaki M. Hara T. Furuya T. Miyazono K. (2002). Two major Smad pathways in TGF-beta superfamily signalling. Genes Cells 7, 1191–1204. 10.1046/j.1365-2443.2002.00599.x, PMID: 12485160

[ref118] Miyazono K. Katsuno Y. Koinuma D. Ehata S. Morikawa M. (2018). Intracellular and extracellular TGF-beta signaling in cancer: some recent topics. Front. Med. 12, 387–411. 10.1007/s11684-018-0646-8, PMID: 30043220

[ref119] Montorfano I. Becerra A. Cerro R. Echeverria C. Saez E. Morales M. G.. (2014). Oxidative stress mediates the conversion of endothelial cells into myofibroblasts via a TGF-beta1 and TGF-beta2-dependent pathway. Lab. Investig. 94, 1068–1082. 10.1038/labinvest.2014.100, PMID: 25068653

[ref120] Ngo H. K. Lee H. G. Piao J. Y. Zhong X. Lee H. N. Han H. J.. (2016). Helicobacter pylori induces snail expression through ROS-mediated activation of Erk and inactivation of GSK-3beta in human gastric cancer cells. Mol. Carcinog. 55, 2236–2246. 10.1002/mc.22464, PMID: 26808296

[ref121] Niranjan R. Thakur A. K. (2017). The toxicological mechanisms of environmental soot (black carbon) and carbon black: focus on oxidative stress and inflammatory pathways. Front. Immunol. 8:763. 10.3389/fimmu.2017.00763, PMID: 28713383 PMC5492873

[ref122] Noakes R. (2015). The aryl hydrocarbon receptor: a review of its role in the physiology and pathology of the integument and its relationship to the tryptophan metabolism. Int. J. Tryptophan Res. 8, 7–18. 10.4137/IJTR.S19985, PMID: 25733915 PMC4327407

[ref123] Oloumi A. McPhee T. Dedhar S. (2004). Regulation of E-cadherin expression and beta-catenin/Tcf transcriptional activity by the integrin-linked kinase. Biochim. Biophys. Acta 1691, 1–15. 10.1016/j.bbamcr.2003.12.002 15053919

[ref124] Oshikawa J. Urao N. Kim H. W. Kaplan N. Razvi M. McKinney R.. (2010). Extracellular SOD-derived H2O2 promotes VEGF signaling in caveolae/lipid rafts and post-ischemic angiogenesis in mice. PLoS One 5:e10189. 10.1371/journal.pone.0010189, PMID: 20422004 PMC2858087

[ref125] Pain M. Bermudez O. Lacoste P. Royer P. J. Botturi K. Tissot A.. (2014). Tissue remodelling in chronic bronchial diseases: from the epithelial to mesenchymal phenotype. Eur. Respir. Rev. 23, 118–130. 10.1183/09059180.00004413, PMID: 24591669 PMC9487272

[ref126] Palleschi S. Rossi B. Armiento G. Montereali M. R. Nardi E. Mazziotti Tagliani S.. (2018). Toxicity of the readily leachable fraction of urban PM_2.5_ to human lung epithelial cells: role of soluble metals. Chemosphere 196, 35–44. 10.1016/j.chemosphere.2017.12.147, PMID: 29289849

[ref127] Qi Z. Song Y. Ding Q. Liao X. Li R. Liu G.. (2019). Water soluble and insoluble components of PM_2.5_ and their functional cardiotoxicities on neonatal rat cardiomyocytes *in vitro*. Ecotoxicol. Environ. Saf. 168, 378–387. 10.1016/j.ecoenv.2018.10.107, PMID: 30396134

[ref128] Qin G. Xia J. Zhang Y. Guo L. Chen R. Sang N. (2018). Ambient fine particulate matter exposure induces reversible cardiac dysfunction and fibrosis in juvenile and older female mice. Part. Fibre Toxicol. 15:27. 10.1186/s12989-018-0264-2, PMID: 29941001 PMC6019275

[ref129] Qiu Y. N. Wang G. H. Zhou F. Hao J. J. Tian L. Guan L. F.. (2019). PM_2.5_ induces liver fibrosis via triggering ROS-mediated mitophagy. Ecotoxicol. Environ. Saf. 167, 178–187. 10.1016/j.ecoenv.2018.08.050, PMID: 30336408

[ref130] Randi A. S. Sanchez M. S. Alvarez L. Cardozo J. Pontillo C. Kleiman de Pisarev D. L. (2008). Hexachlorobenzene triggers AhR translocation to the nucleus, c-Src activation and EGFR transactivation in rat liver. Toxicol. Lett. 177, 116–122. 10.1016/j.toxlet.2008.01.003, PMID: 18295415

[ref131] Rhyu D. Y. Yang Y. Ha H. Lee G. T. Song J. S. Uh S. T.. (2005). Role of reactive oxygen species in TGF-beta1-induced mitogen-activated protein kinase activation and epithelial-mesenchymal transition in renal tubular epithelial cells. J. Am. Soc. Nephrol. 16, 667–675. 10.1681/ASN.2004050425, PMID: 15677311

[ref132] Risom L. Moller P. Loft S. (2005). Oxidative stress-induced DNA damage by particulate air pollution. Mutat. Res. 592, 119–137. 10.1016/j.mrfmmm.2005.06.012 16085126

[ref133] Rynning I. Neca J. Vrbova K. Libalova H. Rossner P. Jr. Holme J. A.. (2018). *In vitro* transformation of human bronchial epithelial cells by diesel exhaust particles: gene expression profiling and early toxic responses. Toxicol. Sci. 166, 51–64. 10.1093/toxsci/kfy183, PMID: 30010986 PMC6204768

[ref134] Safe S. Lee S. O. Jin U. H. (2013). Role of the aryl hydrocarbon receptor in carcinogenesis and potential as a drug target. Toxicol. Sci. 135, 1–16. 10.1093/toxsci/kft128, PMID: 23771949 PMC3748760

[ref135] Saha P. Johny E. Dangi A. Shinde S. Brake S. Eapen M. S.. (2018). Impact of maternal air pollution exposure on children's lung health: an indian perspective. Toxics 6:68. 10.3390/toxics6040068, PMID: 30453488 PMC6315719

[ref136] Sanchez-Tillo E. de Barrios O. Siles L. Cuatrecasas M. Castells A. Postigo A. (2011). Beta-catenin/TCF4 complex induces the epithelial-to-mesenchymal transition (EMT)-activator ZEB1 to regulate tumor invasiveness. Proc. Natl. Acad. Sci. USA 108, 19204–19209. 10.1073/pnas.1108977108 22080605 PMC3228467

[ref137] Santamaria P. G. Moreno-Bueno G. Cano A. (2019). Contribution of epithelial plasticity to therapy resistance. J. Clin. Med. 8:676. 10.3390/jcm8050676, PMID: 31091749 PMC6571660

[ref138] Santiago L. Daniels G. Wang D. Deng F. M. Lee P. (2017). Wnt signaling pathway protein LEF1 in cancer, as a biomarker for prognosis and a target for treatment. Am. J. Cancer Res. 7, 1389–1406. PMID: 28670499 PMC5489786

[ref139] Sanyal S. Rochereau T. Maesano C. N. Com-Ruelle L. Annesi-Maesano I. (2018). Long-term effect of outdoor air pollution on mortality and morbidity: a 12-year follow-up study for metropolitan France. Int. J. Environ. Res. Public Health 15, 1–8. 10.3390/ijerph15112487, PMID: 30412999 PMC6266056

[ref140] Sarkar F. H. Li Y. Wang Z. Kong D. (2008). NF-kappaB signaling pathway and its therapeutic implications in human diseases. Int. Rev. Immunol. 27, 293–319. 10.1080/08830180802276179 18853341

[ref141] Sattayakhom A. Chunglok W. Ittarat W. Chamulitrat W. (2014). Study designs to investigate Nox1 acceleration of neoplastic progression in immortalized human epithelial cells by selection of differentiation resistant cells. Redox Biol. 2, 140–147. 10.1016/j.redox.2013.12.010, PMID: 24494188 PMC3909263

[ref142] Sayin V. I. Ibrahim M. X. Larsson E. Nilsson J. A. Lindahl P. Bergo M. O. (2014). Antioxidants accelerate lung cancer progression in mice. Sci. Transl. Med. 6:221ra215. 10.1126/scitranslmed.3007653, PMID: 24477002

[ref143] Schraufnagel D. E. Balmes J. Cowl C. T. De Matteis S. Jung S. H. Mortimer K. (2018). Air pollution and non-communicable diseases: a review by the forum of international respiratory societies' environmental committee, part 1: the damaging effects of air pollution. Chest 155, 409–416. 10.1016/j.chest.2018.10.042 30419235 PMC6904855

[ref144] Sese L. Nunes H. Cottin V. Sanyal S. Didier M. Carton Z.. (2018). Role of atmospheric pollution on the natural history of idiopathic pulmonary fibrosis. Thorax 73, 145–150. 10.1136/thoraxjnl-2017-209967, PMID: 28798214

[ref145] Shi Y. Zhao T. Yang X. Sun B. Li Y. Duan J. (2019). PM_2.5_-induced alteration of DNA methylation and RNA-transcription are associated with inflammatory response and lung injury. Sci. Total Environ. 650, 908–921. 10.1016/j.scitotenv.2018.09.085 30308865

[ref146] Shimba S. Komiyama K. Moro I. Tezuka M. (2002). Overexpression of the aryl hydrocarbon receptor (AhR) accelerates the cell proliferation of A549 cells. J. Biochem. 132, 795–802. 10.1093/oxfordjournals.jbchem.a003289, PMID: 12417031

[ref147] Shu D. Y. Wojciechowski M. Lovicu F. J. (2018). ERK1/2-mediated EGFR-signaling is required for TGFbeta-induced lens epithelial-mesenchymal transition. Exp. Eye Res. 178, 108–121. 10.1016/j.exer.2018.09.021 30290164

[ref148] Shukla A. Timblin C. BeruBe K. Gordon T. McKinney W. Driscoll K.. (2000). Inhaled particulate matter causes expression of nuclear factor (NF)-kappaB-related genes and oxidant-dependent NF-kappaB activation *in vitro*. Am. J. Respir. Cell Mol. Biol. 23, 182–187. 10.1165/ajrcmb.23.2.4035, PMID: 10919984

[ref149] Shuster-Meiseles T. Shafer M. M. Heo J. Pardo M. Antkiewicz D. S. Schauer J. J.. (2016). ROS-generating/ARE-activating capacity of metals in roadway particulate matter deposited in urban environment. Environ. Res. 146, 252–262. 10.1016/j.envres.2016.01.009, PMID: 26775006

[ref150] Simeone P. Trerotola M. Franck J. Cardon T. Marchisio M. Fournier I. (2018). The multiverse nature of epithelial to mesenchymal transition. Semin. Cancer Biol. 58, 1–10. 10.1016/j.semcancer.2018.11.004 30453041

[ref151] Song L. Li D. Li X. Ma L. Bai X. Wen Z.. (2017). Exposure to PM_2.5_ induces aberrant activation of NF-kappaB in human airway epithelial cells by downregulating miR-331 expression. Environ. Toxicol. Pharmacol. 50, 192–199. 10.1016/j.etap.2017.02.011, PMID: 28192748

[ref152] Squadrito G. L. Cueto R. Dellinger B. Pryor W. A. (2001). Quinoid redox cycling as a mechanism for sustained free radical generation by inhaled airborne particulate matter. Free Radic. Biol. Med. 31, 1132–1138. 10.1016/S0891-5849(01)00703-1, PMID: 11677046

[ref153] Stewart T. A. Azimi I. Thompson E. W. Roberts-Thomson S. J. Monteith G. R. (2015). A role for calcium in the regulation of ATP-binding cassette, sub-family C, member 3 (ABCC3) gene expression in a model of epidermal growth factor-mediated breast cancer epithelial-mesenchymal transition. Biochem. Biophys. Res. Commun. 458, 509–514. 10.1016/j.bbrc.2015.01.141, PMID: 25666946

[ref154] Stone R. C. Pastar I. Ojeh N. Chen V. Liu S. Garzon K. I.. (2016). Epithelial-mesenchymal transition in tissue repair and fibrosis. Cell Tissue Res. 365, 495–506. 10.1007/s00441-016-2464-0, PMID: 27461257 PMC5011038

[ref155] Storci G. Sansone P. Mari S. D'Uva G. Tavolari S. Guarnieri T.. (2010). TNFalpha up-regulates SLUG via the NF-kappaB/HIF1alpha axis, which imparts breast cancer cells with a stem cell-like phenotype. J. Cell. Physiol. 225, 682–691. 10.1002/jcp.22264, PMID: 20509143 PMC2939957

[ref156] Sullivan N. J. Sasser A. K. Axel A. E. Vesuna F. Raman V. Ramirez N.. (2009). Interleukin-6 induces an epithelial-mesenchymal transition phenotype in human breast cancer cells. Oncogene 28, 2940–2947. 10.1038/onc.2009.180, PMID: 19581928 PMC5576031

[ref157] Sun J. Shen Z. Zeng Y. Niu X. Wang J. Cao J.. (2018). Characterization and cytotoxicity of PAHs in PM_2.5_ emitted from residential solid fuel burning in the Guanzhong plain, China. Environ. Pollut. 241, 359–368. 10.1016/j.envpol.2018.05.076, PMID: 29852439

[ref520] Sun B. Shi Y. Li Y. Jiang J. Liang S. Duan J.. (2019). Short-term PM2.5 exposure induces sustained pulmonary fibrosis development during post-exposure period in rats. J. Hazard Mater. 1–11. 10.1016/j.jhazmat.2019.121566 (in press). PMID: 31761645

[ref158] Sun S. Xie F. Zhang Q. Cui Z. Cheng X. Zhong F.. (2017). Advanced oxidation protein products induce hepatocyte epithelial-mesenchymal transition via a ROS-dependent, TGF-beta/Smad signaling pathway. Cell Biol. Int. 41, 842–853. 10.1002/cbin.10792, PMID: 28500745

[ref159] Tang W. Du L. Sun W. Yu Z. He F. Chen J.. (2017). Maternal exposure to fine particulate air pollution induces epithelial-to-mesenchymal transition resulting in postnatal pulmonary dysfunction mediated by transforming growth factor-beta/Smad3 signaling. Toxicol. Lett. 267, 11–20. 10.1016/j.toxlet.2016.12.016, PMID: 28041981

[ref160] Taube J. H. Herschkowitz J. I. Komurov K. Zhou A. Y. Gupta S. Yang J. (2010). Core epithelial-to-mesenchymal transition interactome gene-expression signature is associated with claudin-low and metaplastic breast cancer subtypes. Proc. Natl. Acad. Sci. USA 107, 15449–15454. 10.1073/pnas.1004900107 20713713 PMC2932589

[ref161] Techasen A. Namwat N. Loilome W. Bungkanjana P. Khuntikeo N. Puapairoj A.. (2012). Tumor necrosis factor-alpha (TNF-alpha) stimulates the epithelial-mesenchymal transition regulator snail in cholangiocarcinoma. Med. Oncol. 29, 3083–3091. 10.1007/s12032-012-0305-x, PMID: 22903530

[ref162] Thevenot P. T. Saravia J. Jin N. Giaimo J. D. Chustz R. E. Mahne S.. (2013). Radical-containing ultrafine particulate matter initiates epithelial-to-mesenchymal transitions in airway epithelial cells. Am. J. Respir. Cell Mol. Biol. 48, 188–197. 10.1165/rcmb.2012-0052OC, PMID: 23087054 PMC3604062

[ref163] Thiery J. P. Acloque H. Huang R. Y. Nieto M. A. (2009). Epithelial-mesenchymal transitions in development and disease. Cell 139, 871–890. 10.1016/j.cell.2009.11.007, PMID: 19945376

[ref164] Thuault S. Tan E. J. Peinado H. Cano A. Heldin C. H. Moustakas A. (2008). HMGA2 and Smads co-regulate SNAIL1 expression during induction of epithelial-to-mesenchymal transition. J. Biol. Chem. 283, 33437–33446. 10.1074/jbc.M802016200, PMID: 18832382 PMC2662269

[ref165] Thuault S. Valcourt U. Petersen M. Manfioletti G. Heldin C. H. Moustakas A. (2006). Transforming growth factor-beta employs HMGA2 to elicit epithelial-mesenchymal transition. J. Cell Biol. 174, 175–183. 10.1083/jcb.200512110, PMID: 16831886 PMC2064178

[ref166] Tripathi P. Deng F. Scruggs A. M. Chen Y. Huang S. K. (2018). Variation in doses and duration of particulate matter exposure in bronchial epithelial cells results in upregulation of different genes associated with airway disorders. Toxicol. In Vitro 51, 95–105. 10.1016/j.tiv.2018.05.004, PMID: 29753051 PMC6464127

[ref167] Tripathi K. Garg M. (2018). Mechanistic regulation of epithelial-to-mesenchymal transition through RAS signaling pathway and therapeutic implications in human cancer. J. Cell Commun. Signal. 12, 513–527. 10.1007/s12079-017-0441-3, PMID: 29330773 PMC6039341

[ref168] Valavanidis A. Vlachogianni T. Fiotakis K. Loridas S. (2013). Pulmonary oxidative stress, inflammation and cancer: respirable particulate matter, fibrous dusts and ozone as major causes of lung carcinogenesis through reactive oxygen species mechanisms. Int. J. Environ. Res. Public Health 10, 3886–3907. 10.3390/ijerph10093886, PMID: 23985773 PMC3799517

[ref169] Vander Ark A. Cao J. Li X. (2018). TGF-beta receptors: in and beyond TGF-beta signaling. Cell. Signal. 52, 112–120. 10.1016/j.cellsig.2018.09.002, PMID: 30184463

[ref170] Vattanasit U. Navasumrit P. Khadka M. B. Kanitwithayanun J. Promvijit J. Autrup H.. (2014). Oxidative DNA damage and inflammatory responses in cultured human cells and in humans exposed to traffic-related particles. Int. J. Hyg. Environ. Health 217, 23–33. 10.1016/j.ijheh.2013.03.002, PMID: 23567252

[ref171] Vesuna F. van Diest P. Chen J. H. Raman V. (2008). Twist is a transcriptional repressor of E-cadherin gene expression in breast cancer. Biochem. Biophys. Res. Commun. 367, 235–241. 10.1016/j.bbrc.2007.11.151, PMID: 18062917 PMC2696127

[ref172] Vinson K. E. George D. C. Fender A. W. Bertrand F. E. Sigounas G. (2016). The notch pathway in colorectal cancer. Int. J. Cancer 138, 1835–1842. 10.1002/ijc.29800, PMID: 26264352

[ref173] Wan Q. Liu Z. Yang Y. (2018). Puerarin inhibits vascular smooth muscle cells proliferation induced by fine particulate matter via suppressing of the p38 MAPK signaling pathway. BMC Complement. Altern. Med. 18:146. 10.1186/s12906-018-2206-9, PMID: 29728095 PMC5935934

[ref174] Wang C. K. Chang H. Chen P. H. Chang J. T. Kuo Y. C. Ko J. L.. (2009). Aryl hydrocarbon receptor activation and overexpression upregulated fibroblast growth factor-9 in human lung adenocarcinomas. Int. J. Cancer 125, 807–815. 10.1002/ijc.24348, PMID: 19358281

[ref175] Wang Y. Sun B. Zhao X. Zhao N. Sun R. Zhu D.. (2016). Twist1-related miR-26b-5p suppresses epithelial-mesenchymal transition, migration and invasion by targeting SMAD1 in hepatocellular carcinoma. Oncotarget 7, 24383–24401. 10.18632/oncotarget.8328, PMID: 27027434 PMC5029709

[ref176] Wang Y. Xu M. Ke Z. J. Luo J. (2017). Cellular and molecular mechanisms underlying alcohol-induced aggressiveness of breast cancer. Pharmacol. Res. 115, 299–308. 10.1016/j.phrs.2016.12.005, PMID: 27939360 PMC5205572

[ref177] Wang J. Zhang W. J. Xiong W. Lu W. H. Zheng H. Y. Zhou X. (2018). PM_2.5_ stimulated the release of cytokines from BEAS-2B cells through activation of IKK/NF-kappaB pathway. Hum. Exp. Toxicol. 38, 311–320. 10.1177/0960327118802628 30354488

[ref178] Wang G. Zhen L. Lu P. Jiang R. Song W. (2013). Effects of ozone and fine particulate matter (PM_2.5_) on rat cardiac autonomic nervous system and systemic inflammation. Wei Sheng Yan Jiu 42, 554–560. PMID: 24024363

[ref179] Wang Y. Zhong Y. Hou T. Liao J. Zhang C. Sun C.. (2019). PM_2.5_ induces EMT and promotes CSC properties by activating notch pathway in vivo and vitro. Ecotoxicol. Environ. Saf. 178, 159–167. 10.1016/j.ecoenv.2019.03.086, PMID: 31002970

[ref180] Wei H. Liang F. Cheng W. Zhou R. Wu X. Feng Y.. (2017). The mechanisms for lung cancer risk of PM_2.5_: induction of epithelial-mesenchymal transition and cancer stem cell properties in human non-small cell lung cancer cells. Environ. Toxicol. 32, 2341–2351. 10.1002/tox.22437, PMID: 28846189

[ref181] Wu W. S. (2006). The signaling mechanism of ROS in tumor progression. Cancer Metastasis Rev. 25, 695–705. 10.1007/s10555-006-9037-8, PMID: 17160708

[ref182] Xie Y. Zhao B. (2018). Chemical composition of outdoor and indoor PM_2.5_ collected during haze events: transformations and modified source contributions resulting from outdoor-to-indoor transport. Indoor Air 28, 828–839. 10.1111/ina.12503, PMID: 30156041

[ref183] Xin L. Che B. Zhai B. Luo Q. Zhang C. Wang J. (2018). 1,25-Dihydroxy vitamin D3 attenuates the oxidative stress-mediated inflammation induced by PM_2.5_ via the p38/NF-kappaB/NLRP3 pathway. Inflammation 42, 702–713. 10.1007/s10753-018-0928-y 30430362

[ref184] Xu H. Jiao X. Wu Y. Li S. Cao L. Dong L. (2018). Exosomes derived from PM_2.5_ treated lung cancer cells promote the growth of lung cancer via the Wnt3a/betacatenin pathway. Oncol. Rep. 41, 1180–1188. 10.3892/or.2018.6862 30431139

[ref185] Xu Z. Li Z. Liao Z. Gao S. Hua L. Ye X. (2019a). PM_2.5_ induced pulmonary fibrosis *in vivo* and *in vitro*. Ecotoxicol. Environ. Saf. 171, 112–121. 10.1016/j.ecoenv.2018.12.061 30597315

[ref186] Xu Z. Wang N. Xu Y. Hua L. Zhou D. Zheng M. (2019b). Effects of chronic PM_2.5_ exposure on pulmonary epithelia: transcriptome analysis of mRNA-exosomal miRNA interactions. Toxicol. Lett. 316, 49–59. 10.1016/j.toxlet.2019.09.010 31520698

[ref187] Xu Z. Zhang Z. Ma X. Ping F. Zheng X. (2015). Effect of PM_2.5_ on oxidative stress-JAK/STAT signaling pathway of human bronchial epithelial cells. Wei Sheng Yan Jiu 44, 451–455. PMID: 26137628

[ref188] Yadav A. Kumar B. Datta J. Teknos T. N. Kumar P. (2011). IL-6 promotes head and neck tumor metastasis by inducing epithelial-mesenchymal transition via the JAK-STAT3-SNAIL signaling pathway. Mol. Cancer Res. 9, 1658–1667. 10.1158/1541-7786.MCR-11-0271, PMID: 21976712 PMC3243808

[ref189] Yamaoka T. Kusumoto S. Ando K. Ohba M. Ohmori T. (2018). Receptor tyrosine kinase-targeted cancer therapy. Int. J. Mol. Sci. 19:3491. 10.3390/ijms19113491, PMID: 30404198 PMC6274851

[ref190] Yan Z. Jin Y. An Z. Liu Y. Samet J. M. Wu W. (2016). Inflammatory cell signaling following exposures to particulate matter and ozone. Biochim. Biophys. Acta 1860, 2826–2834. 10.1016/j.bbagen.2016.03.030 27015762

[ref191] Yan L. Xu F. Dai C. L. (2018). Relationship between epithelial-to-mesenchymal transition and the inflammatory microenvironment of hepatocellular carcinoma. J. Exp. Clin. Cancer Res. 37:203. 10.1186/s13046-018-0887-z, PMID: 30157906 PMC6114477

[ref192] Yang L. Lin Z. Wang Y. Li C. Xu W. Li Q.. (2018). Nickle(II) ions exacerbate bleomycin-induced pulmonary inflammation and fibrosis by activating the ROS/Akt signaling pathway. Environ. Sci. Pollut. Res. Int. 25, 4406–4418. 10.1007/s11356-017-0525-x, PMID: 29185215

[ref193] Yang D. Ma M. Zhou W. Yang B. Xiao C. (2017). Inhibition of miR-32 activity promoted EMT induced by PM_2.5_ exposure through the modulation of the Smad1-mediated signaling pathways in lung cancer cells. Chemosphere 184, 289–298. 10.1016/j.chemosphere.2017.05.152, PMID: 28601662

[ref194] Yang M. C. Wang H. C. Hou Y. C. Tung H. L. Chiu T. J. Shan Y. S. (2015). Blockade of autophagy reduces pancreatic cancer stem cell activity and potentiates the tumoricidal effect of gemcitabine. Mol. Cancer 14:179. 10.1186/s12943-015-0449-3, PMID: 26458814 PMC4603764

[ref195] Ye X. Hong W. Hao B. Peng G. Huang L. Zhao Z.. (2018). PM_2.5_ promotes human bronchial smooth muscle cell migration via the sonic hedgehog signaling pathway. Respir. Res. 19:37. 10.1186/s12931-017-0702-y, PMID: 29499705 PMC5833105

[ref196] Yi L. Wei C. Fan W. (2017). Fine-particulate matter (PM_2.5_), a risk factor for rat gestational diabetes with altered blood glucose and pancreatic GLUT2 expression. Gynecol. Endocrinol. 33, 611–616. 10.1080/09513590.2017.1301923, PMID: 28368218

[ref197] Yilmaz M. Christofori G. (2009). EMT, the cytoskeleton, and cancer cell invasion. Cancer Metastasis Rev. 28, 15–33. 10.1007/s10555-008-9169-0, PMID: 19169796

[ref198] Yuan Q. Chen Y. Li X. Zhang Z. Chu H. (2019). Ambient fine particulate matter (PM_2.5_) induces oxidative stress and pro-inflammatory response via up-regulating the expression of CYP1A1/1B1 in human bronchial epithelial cells *in vitro*. Mutat. Res. Genet. Toxicol. Environ. Mutagen. 839, 40–48. 10.1016/j.mrgentox.2018.12.005, PMID: 30744811

[ref199] Zavadil J. Bottinger E. P. (2005). TGF-beta and epithelial-to-mesenchymal transitions. Oncogene 24, 5764–5774. 10.1038/sj.onc.1208927, PMID: 16123809

[ref200] Zhang X. Cheng Q. Yin H. Yang G. (2017b). Regulation of autophagy and EMT by the interplay between p53 and RAS during cancer progression (review). Int. J. Oncol. 51, 18–24. 10.3892/ijo.2017.4025 28560457

[ref201] Zhang H. Li Z. (2019). microRNA-16 via Twist1 inhibits EMT induced by PM_2.5_ exposure in human hepatocellular carcinoma. Open Med. 14, 673–682. 10.1515/med-2019-0078, PMID: 31572802 PMC6749726

[ref202] Zhang Q. Luo Q. Yuan X. Chai L. Li D. Liu J. (2017a). Atmospheric particulate matter 2.5 promotes the migration and invasion of hepatocellular carcinoma cells. Oncol. Lett. 13, 3445–3450. 10.3892/ol.2017.5947 28521450 PMC5431175

[ref203] Zhang J. Tian X. J. Xing J. (2016a). Signal transduction pathways of EMT induced by TGF-beta, SHH, and WNT and their crosstalks. J. Clin. Med. 5, 1–18. 10.3390/jcm5040041 PMC4850464 27043642

[ref204] Zhang J. Wang X. Vikash V. Ye Q. Wu D. Liu Y. (2016b). ROS and ROS-mediated cellular signaling. Oxidative Med. Cell. Longev. 2016:4350965. 10.1155/2016/4350965 PMC4779832 26998193

[ref205] Zhao H. Tong G. Liu J. Wang J. Zhang H. Bai J.. (2019). IP3R and RyR channels are involved in traffic-related PM_2.5_-induced disorders of calcium homeostasis. Toxicol. Ind. Health 35, 339–348. 10.1177/0748233719843763, PMID: 31023176

[ref206] Zhao Y. Xu Y. Li Y. Xu W. Luo F. Wang B.. (2013). NF-kappaB-mediated inflammation leading to EMT via miR-200c is involved in cell transformation induced by cigarette smoke extract. Toxicol. Sci. 135, 265–276. 10.1093/toxsci/kft150, PMID: 23824089

[ref207] Zheng L. Liu S. Zhuang G. Xu J. Liu Q. Zhang X.. (2017). Signal transductions of BEAS-2B cells in response to carcinogenic PM_2.5_ exposure based on a microfluidic system. Anal. Chem. 89, 5413–5421. 10.1021/acs.analchem.7b00218, PMID: 28447797

[ref208] Zhou B. P. Deng J. Xia W. Xu J. Li Y. M. Gunduz M.. (2004). Dual regulation of snail by GSK-3beta-mediated phosphorylation in control of epithelial-mesenchymal transition. Nat. Cell Biol. 6, 931–940. 10.1038/ncb1173, PMID: 15448698

[ref209] Zou W. He F. Liu S. Pu J. Hu J. Sheng Q.. (2018). PM_2.5_ induced the expression of fibrogenic mediators via HMGB1-RAGE signaling in human airway epithelial cells. Can. Respir. J. 2018:1817398. 10.1155/2018/1817398, PMID: 29670673 PMC5833260

[ref210] Zou X. Z. Liu T. Gong Z. C. Hu C. P. Zhang Z. (2017). MicroRNAs-mediated epithelial-mesenchymal transition in fibrotic diseases. Eur. J. Pharmacol. 796, 190–206. 10.1016/j.ejphar.2016.12.003, PMID: 27916556

